# Engineering a Mesoporous Silicon Nanoparticle Cage to Enhance Performance of a Phosphotriesterase Enzyme for Degradation of VX Nerve Agent

**DOI:** 10.1002/advs.202409535

**Published:** 2024-11-04

**Authors:** Yi‐Sheng Lu, Eduardo Reynoso Moreno, Yubin Huang, Ruhan Fan, Ashley T. Tucker, Linnzi K. Wright, Ronald A. Evans, Brooke M. Ahern, Donald E. Owens, Stephen A. Chappell, Dale J. Christensen, John Dresios, Michael J. Sailor

**Affiliations:** ^1^ Department of Chemistry and Biochemistry University of California San Diego La Jolla CA 92093 USA; ^2^ Leidos 10260 Campus Point Drive San Diego CA 92121 United States; ^3^ Materials Science and Engineering Program University of California San Diego La Jolla CA 92093 USA; ^4^ US Army Combat Capabilities Development Command Chemical Biological Center 8938 N Kings Creek Rd., E3150 Gunpowder MD 21010 USA; ^5^ TFF Pharmaceuticals 1751 River Run Fort Worth TX 76107 USA

**Keywords:** acetylcholinesterase activity assay, dermal protection, enzyme immobilization, enzyme stability, phosphotriesterase variant L7ep‐3a

## Abstract

The organophosphate (OP)‐hydrolyzing enzyme phosphotriesterase (PTE, variant L7ep‐3a) immobilized within a partially oxidized mesoporous silicon nanoparticle cage is synthesized and the catalytic performance of the enzyme@nanoparticle construct for hydrolysis of a simulant, dimethyl p‐nitrophenyl phosphate (DMNP), and the live nerve agent VX is benchmarked against the free enzyme. In a neutral aqueous buffer, the optimized construct shows a ≈2‐fold increase in the rate of DMNP turnover relative to the free enzyme. Enzyme@nanoparticles with more hydrophobic surface chemistry in the interior of the pores show lower catalytic activity, suggesting the importance of hydration of the pore interior on performance. The enzyme@nanoparticle construct is readily separated from the neutralized agent; the nanoparticle is found to retain DMNP hydrolysis activity through seven decontamination/recovery cycles. The nanoparticle cage stabilizes the enzyme against thermal denaturing and enzymatic (trypsin) degradation conditions relative to free enzyme. When incorporated into a topical gel formulation, the PTE‐loaded nanoparticles show high activity toward the nerve agent VX in an ex vivo rabbit skin model. In vitro acetylcholinesterase (AChE) assays in human blood show that the enzyme@nanoparticle construct decontaminates VX, preserving the biological function of AChE when exposed to an otherwise incapacitating dose.

## Graphical abstract

A porous silicon nanocage encapsulating phosphotriesterase variant L7ep‐3a is evaluated for nerve agent detoxification. By tuning the physicochemical properties of the enzyme–porous silicon nanoconstruct, enhanced catalytic activity, and operational stability are achieved compared to the free enzyme. Bolstered by its biocompatibility with human epidermal tissue, the nanobiocatalyst presents a strategy for nerve agent detoxification.

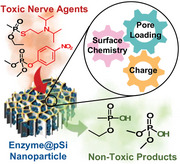

## 1 Introduction

The persistent threat posed by toxic organophosphorus compounds (OPs) across various settings, ranging from military operations to civilian healthcare, remains an ongoing challenge in developing effective neutralization strategies against these hazardous substances.^[^ ^1^, ^2^, ^3^ ^]^ The acute toxicity and rapid symptom onset from OP exposure have fueled tremendous interest in the synthesis of materials capable of catalytically degrading nerve agents (e.g., polymers,^[^ ^4^, ^5^ ^]^ oxime‐functionalized catalysts,^[^ ^6^ ^]^ metal nanoparticles,^[^ ^7^, ^8^ ^]^ and metal‐organic frameworks^[^ ^9^, ^10^, ^11^ ^]^), and their integration into protective garments for first responders,^[^ ^12^, ^13^ ^]^ existing antidotes and treatment methods,^[^ ^14^, ^15^ ^]^ and topical detoxifying barrier lotions or ointments.^[^ ^16^, ^17^ ^]^ The ultimate benchmark for these synthetic catalytic materials is the OP‐degrading enzymes, known for their innate biocompatibility and exceptional efficiency in countering the rapid action of OPs, even under mild reaction conditions.^[^ ^18^, ^19^, ^20^ ^]^ However, a significant limitation of OP‐degrading enzymes lies in their susceptibility to external stresses, limiting their operation to conditions similar to their native environment.^[^ ^18^ ^]^ A complementary approach to further engineering of the enzyme is immobilization of the existing OP‐degrading enzyme within a host material, enhancing the range of operational stability and the reusability of the enzyme,^[^ ^21^, ^22^, ^23^, ^24^, ^25^, ^26^, ^27^ ^]^ and, in some cases, even enhancing activity.^[^ ^28^ ^]^ For in vivo prophylactic treatments against OP poisoning, utilizing a host material can delay clearance by the innate immune system, thereby extending the residence time and functional lifetime of the enzyme.^[^ ^15^, ^29^ ^]^ Despite the growing potential for immobilized OP‐degrading enzymes, their catalytic activity is often attenuated following immobilization within host materials, impeding their practical application.^[^ ^21^, ^22^, ^30^ ^]^

Porous silicon nanoparticles (pSiNPs), with their versatile structural and chemical tunability, provide an attractive host material for preserving the catalytic activity of OP‐degrading enzymes. The mesopore size in the pSiNP nanostructure is readily controlled to a nanometer resolution (2–20 nm) during electrochemical fabrication^[^ ^31^ ^]^ or by post‐synthetic treatments,^[^ ^32^, ^33^, ^34^ ^]^ allowing accommodation of enzymes with sizes spanning the low mesopore regime. This is a unique feature that is not as readily achieved with the more common (and less expensive) silicas derived from sol‐gel routes.^[^ ^35^ ^]^ Underlying this adaptability is the potential to tune the enzyme's physical confinement within the pores of the pSiNP host. By restricting the conformational flexibility of the enzyme, it is less likely to undergo unfolding and denaturation but retains its ability to engage substrates in catalytic reactions.^[^ ^28^ ^]^ The surface chemistry of pSiNPs can be tailored to enable requisite characteristics^[^ ^36^, ^37^, ^38^ ^]^ of the substrate–nanoparticle interface, facilitating substrate association and solvation,^[^ ^39^, ^40^ ^]^ as well as product egress from the encapsulating host.^[^ ^41^, ^42^ ^]^ While other systems have demonstrated similar advantages in immobilizing OP‐degrading enzymes using polymeric matrices,^[^ ^39^, ^40^ ^]^ mesoporous silica,^[^ ^28^ ^]^ metal‐ and covalent‐organic frameworks,^[^ ^22^, ^43^, ^44^ ^]^ they can involve complicated synthetic procedures^[^ ^44^ ^]^ or introduce undesired toxicity, such as leaching of metal ions or framework ligands,^[^ ^45^ ^]^ that reduces their utility in biomedical applications. In contrast, pSiNPs possess well‐established biocompatibility and biodegradability,^[^ ^35^, ^46^ ^]^ making them well suited for enzyme‐inorganic nanocomposites intended for biomedical countermeasures against OPs. Finally, a distinctive feature of electrochemically prepared porous silicon (and porous silicas derived from it) is that it is produced in a non‐equilibrium state,^[^ ^32^, ^33^ ^]^ unlike the more common sol‐gel‐derived materials. This leads to thermodynamically driven restructuring of the material,^[^ ^47^, ^48^ ^]^ which drives the dissolution kinetics^[^ ^49^ ^]^ and enables the encapsulation chemistry used in the present work.

**Scheme 1:**
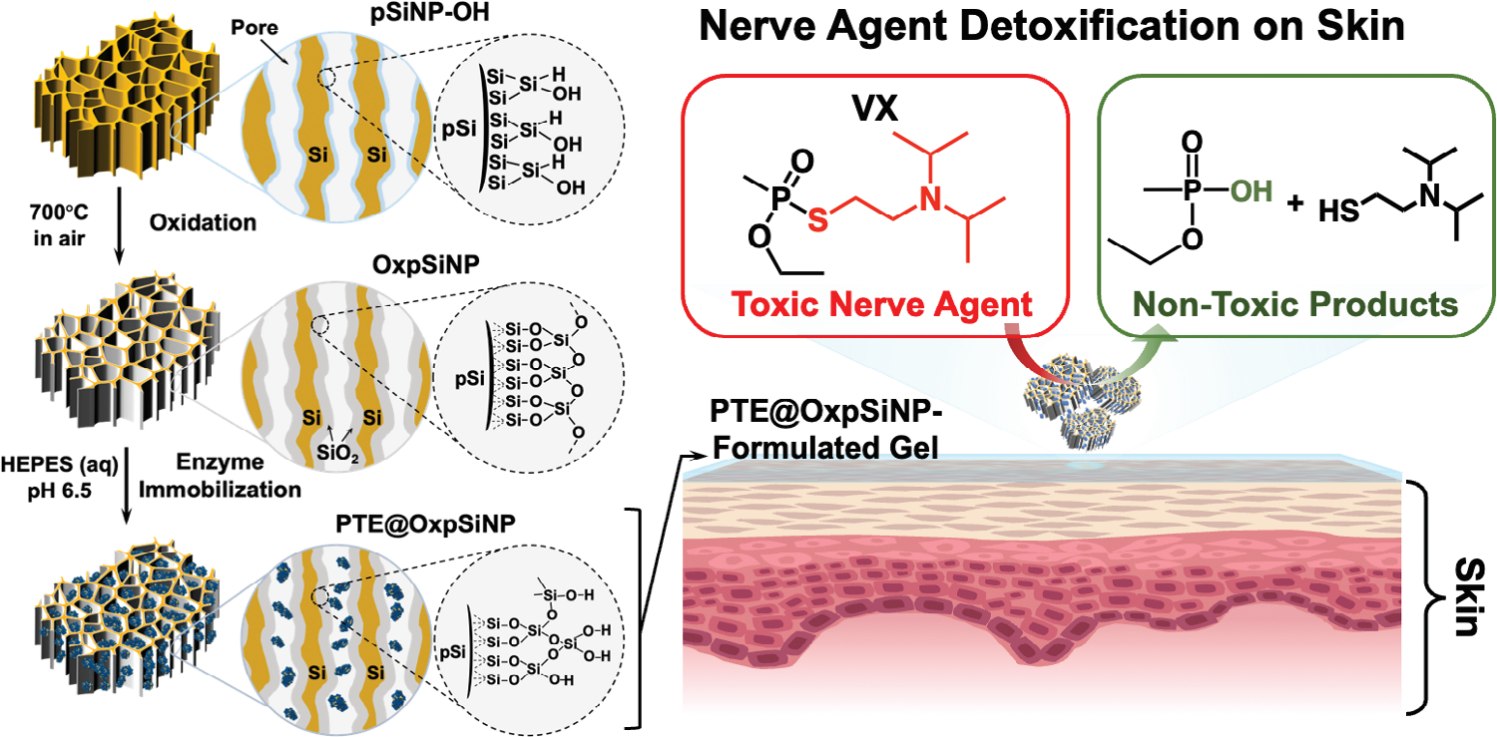
Schematic illustrating the preparation of the PTE@OxpSiNP construct and its potential application as a topical detoxifying agent against VX nerve agent skin exposure. Preparation of the PTE@OxpSiNP construct involves oxidation of as‐formed pSiNP‐OH by thermal oxidation at 700 °C, to form a SiO_2_ shell on the Si framework, OxpSiNPs. The subsequent immobilization of the enzyme L7ep3a into OxpSiNPs is achieved by placing the nanoparticles into a solution of the enzyme in a buffer whose pH matches the enzyme's isoelectric point (pI). The resulting PTE@OxpSiNP construct is integrated into Carbopol gel, providing a skin‐compatible gel capable of catalytically hydrolyzing toxic nerve agent VX into non‐toxic byproducts, ethyl methyl phosphonic acid (EMPA), and 2‐diisopropylaminoethanethiol.

In this study, we address two primary challenges for enzyme encapsulation: 1) increasing catalytic performance, and 2) enabling function in media relevant for in‐field use of an OP‐degrading enzyme. We chose the phosphotriesterase (PTE) variant enzyme L7ep3a as the enzyme of study due to its high hydrolytic activity^[^ ^50^ ^]^ against a wide range of OP compounds, and we demonstrate a potential application of the resulting enzyme‐immobilized pSiNP construct in a skin gel formulation for detoxifying the live nerve agent VX (**Scheme** **1**). Prior work has shown that enzymatic action of PTE on VX generates non‐toxic ethyl methyl phosphonic acid (EMPA) and 2‐diisopropylaminoethanethiol as the hydrolysis products.^[^ ^50^, ^51^ ^]^ For this study, we use thermally oxidized Si‐SiO_2_ core‐shell nanoparticles, which consist of a mesoporous silicon framework encapsulated in a SiO_2_ shell (OxpSiNPs) as the host. Comparative analytical and kinetics studies show that PTE L7ep3a immobilized in OxpSiNPs (PTE@OxpSiNPs) exhibits a greater rate of hydrolysis of the nerve agent simulant, dimethyl *p*‐nitrophenyl phosphate (DMNP) than the free enzyme, and the material is benchmarked against control PTE‐immobilized pSiNP constructs. We show that the optimized PTE@OxpSiNP construct is reusable, and it displays enhanced stability when subjected to elevated temperatures and the proteolytic protein trypsin, outperforming the free enzyme counterpart. Finally, assessments using an ex vivo rabbit skin model and acetylcholinesterase (AChE) inhibition assay demonstrate that the PTE@OxpSiNPs can be integrated into a topical gel formulation effective in detoxifying nerve agent VX and mitigating potential toxicity through skin exposure (Scheme 1).

## 2 Results and Discussion

### 2.1 Preparation and Characterization of the PTE@OxpSiNP Construct

The preparation of the PTE enzyme‐loaded thermally oxidized porous silicon nanoparticle (PTE@OxpSiNP) construct is delineated in Scheme 1. The starting material, pSiNP‐OH, was prepared following the previously published “electrochemical perforation etch” procedure, involving pulsed electrochemical anodization of single crystal silicon wafers to generate a stratified porous silicon layer, followed by removal of the porous layer and ultrasonic fracturing in water at ambient temperature to yield the nanosized particles.^[^ ^31^ ^]^ Because it was only subjected to mild oxidizing conditions, this surface displayed a mixed Si─H and Si─OH surface based on infrared spectroscopic measurements (Figure S1, Supporting Information). In prior work, we found that the mesopore structure of pSiNPs‐OH undergoes an aqueous restructuring process capable of constricting the pores and physically entrapping an enzyme payload.^[^ ^52^ ^]^ While it enhanced enzyme stability, this immobilization chemistry concurrently limited substrate accessibility, reducing the overall catalytic activity of the immobilized enzyme. In the present system, the pSiNPs‐OH sample was thermally oxidized at 700 °C in the air to induce the growth of a thicker, dehydrated silicon dioxide (SiO_2_) shell on the pore walls, generating a type of Si─SiO_2_ core‐shell structure (OxpSiNP). The resulting structures displayed a BET surface area of 223 ± 4 m^2^ g^−1^, and a mean pore diameter of 19 ± 2 nm (Table S1 and Figure S2, Supporting Information). At the 700 °C treatment temperature, which is below the softening point of SiO_2_, silicon nanostructures experience strain due to the lattice mismatch and the difference in coefficient of thermal expansion between the underlying Si nanostructure and the SiO_2_ overlayer.^[^ ^53^, ^54^, ^55^ ^]^ We reasoned that this built‐in strain, along with the highly dehydrated nature of the SiO_2_ overlayer,^[^ ^49^ ^]^ would provide a driving force for local hydrolytic dissolution^[^ ^32^, ^33^ ^]^ and restructuring^[^ ^47^, ^48^ ^]^ of the surface when exposed to aqueous conditions. This strategy aimed to better preserve the activity of the OP‐degrading enzyme when immobilized in the nanoparticle host, pivotal for its intended application in the catalytic degradation of nerve agents.

**Figure 1:**
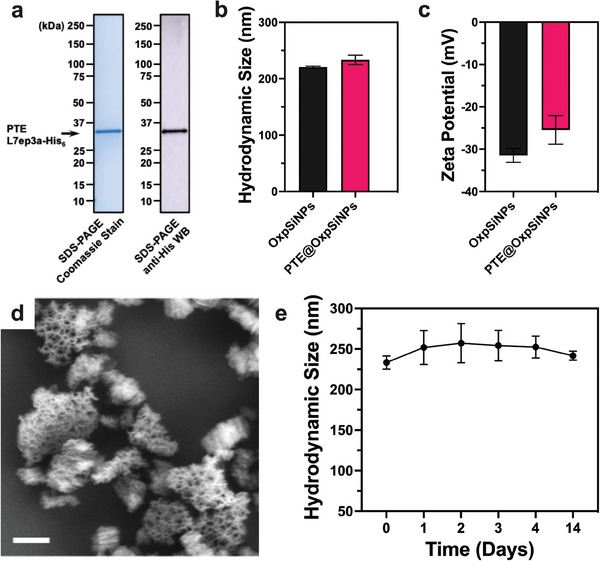
Characterization of PTE L7ep3a‐immobilized nanoparticles (PTE@OxpSiNPs). (a) SDS‐PAGE Coomassie gel staining (left) and anti‐His Western blot (right) of the PTE L7ep3a‐His_6_ enzyme. (b) Mean hydrodynamic diameter and (c) zeta potential of PTE@OxpSiNPs, compared with the non‐loaded OxpSiNPs in HEPES buffer (50 mm, pH 7.4 with 0.1 mm CoCl_2_), measured via dynamic light scattering (DLS). (d) Representative scanning electron microscope (SEM) image of OxpSiNPs after the immobilization of PTE L7ep3a (the PTE@OxpSiNPs). Scale bar: 200 nm. (f) The measured hydrodynamic size of PTE@OxpSiNPs maintained in HEPES buffer (50 mm, pH 7.4 with 0.1 mm CoCl_2_) at room temperature over a 14‐day period. Error bars represent the standard deviation from three independent measurements of three independently prepared samples.

The enzyme PTE (variant L7ep3a, molar mass 36 kDa) was expressed and purified as a hexahistidine–tagged (His_6_–tag) fusion protein in a laboratory strain of Escherichia coli BL21(DE3) following a previously reported protocol.^[^ ^50^ ^]^ Subsequent extraction and purification via nickel nitrilotriacetic acid (Ni‐NTA) chromatography ensured enzyme integrity and purity, verified by sodium dodecyl sulfate‐polyacrylamide gel electrophoresis (SDS‐PAGE) and anti‐His western blotting (**Figure** **1**a). Further experimental details can be found in the Supporting Information. The enzyme was immobilized in the OxpSiNPs by adding the nanoparticles to an aqueous solution containing the enzyme (70 µg mL^−1^) in a buffer (50 mm HEPES, containing 0.1 mm CoCl_2_) set to a pH equal to the enzyme's isoelectric point (pH = pI = 6.5). This pH was chosen to minimize electrostatic repulsions between the enzymes adsorbed on the particle surface and those in the bulk solution, and to maximize adsorption of the protein to the negatively charged SiO_2_ surface, thereby facilitating efficient loading of enzyme into the nanoparticle pores.^[^ ^56^ ^]^ The typical mass loading of the enzyme in the nanoparticles was 5.7 ± 0.6 wt% (mass of loaded enzyme relative to the mass of final enzyme@nanoparticle construct). This mass loading corresponds to a final effective concentration of enzyme within the nanoparticle (mass of enzyme divided by the total apparent volume of the nanoparticle) of 28 mg mL^−1^. Thus the loading procedure effectively concentrated the enzyme into the particles by a factor of 400 relative to the concentration of the “free” enzyme in the loading solution.

In order to investigate the extent to which PTE L7ep3a could infiltrate into the interior pores of the OxpSiNPs, we performed an enzyme loading experiment on larger microparticles of the same composition and pore dimensions as the OxpSiNP material. The larger particles allowed the use of confocal scanning laser microscopy (CSLM) to resolve the enzyme distribution within the mesoporous material. The enzyme was labeled with fluorescein N‐hydroxysuccinimide (FAM‐NHS, emitting green fluorescence) to enable visualization of the spatial distribution by CSLM imaging (Figure S3a, Supporting Information). Quantitative analysis of the fluorescence intensity along the z‐direction of the CLSM images revealed that enzyme penetrated and deposited throughout the microparticles (Figure S3b, Supporting Information). This data suggest that enzyme can be expected to distribute throughout the interior of the nanoparticle hosts as well.

With the loading of enzyme confirmed, we next quantified the average size and morphology of the PTE@OxpSiNPs. Dynamic light scattering (DLS) measurements revealed a mean hydrodynamic particle size of 233 ± 8 nm and a zeta potential of −25.5 ± 3.4 mV when measured in a neutral HEPES buffer (50 mm, pH 7.4 with 0.1 mm CoCl_2_, Figure 1b,c). This represented a slight increase (<15 nm) in mean particle size of the PTE@OxpSiNPs relative to the empty OxpSiNPs. Scanning electron microscope (SEM) images showed that the particles retained an open pore morphology with no major structural changes post‐encapsulation (Figure 1d). The PTE@OxpSiNPs showed no significant change to their average size and no observed particle aggregation when stored in a similar buffer for 14 days at room temperature (Figure 1e).

### 2.2 DMNP Hydrolysis Kinetics of PTE@OxpSiNPs

**Figure 2:**
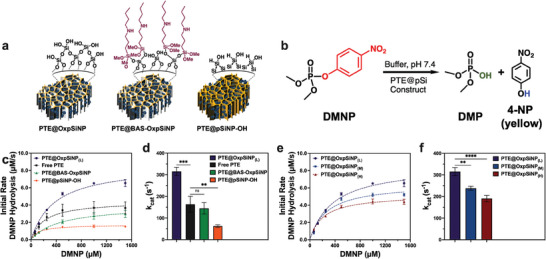
Kinetics of DMNP hydrolysis by PTE@OxpSiNP construct compared to control PTE‐immobilized constructs. (a) Schematic representation of the enzyme@nanoparticle constructs, showing their surface chemistry. (b) Illustration of catalytic hydrolysis of DMNP in the presence of PTE@pSi construct, generating dimethyl phosphate (DMP) and 4‐nitrophenol (4‐NP). 4‐NP exhibits a yellow color under neutral buffer conditions that are readily quantified by optical absorbance measurements. (c) Plot depicting initial rates of conversion of DMNP to 4‐nitrophenol at various concentrations (125–1500 µm) of DMNP, and (d) corresponding kcat values determined for PTE@OxpSiNP, free PTE, PTE@BAS‐OxpSiNP, and PTE@pSiNP‐OH. (e) Plot showing initial rates of conversion of DMNP to 4‐nitrophenol at concentrations (125–1500 µm) of DMNP, and (f) corresponding kcat values for PTE@OxpSiNP with low (L), medium (M), and high (H) mass loading of the PTE L7ep3a enzyme. The mass loading percentage of enzyme for the L, M, and H samples were ≈6, 10, and 15%, respectively. The mass of nanoparticles added was adjusted to maintain a constant total amount of enzyme in each experiment, at an effective PTE concentration of 1 µg mL^−1^ (27.4 nm). Experiments were performed in HEPES buffer (50 mm, pH 7.4 with 0.1 mm CoCl2) at room temperature. Error bars represent standard deviations from three independent measurements and preparations. A one‐way ANOVA test was used for the data (^**^ *p* < 0.01, ^***^ *p* < 0.001, ^****^ *p* < 0.0001; n.s., not significant).

The PTE@OxpSiNPs were compared with control formulations (**Figure** **2**a) for their efficacy in the organophosphorus hydrolysis reaction. The commonly studied simulant dimethyl *p*‐nitrophenyl phosphate (DMNP, Figure 2b) was used as the substrate for these studies, and rates of hydrolysis were compared with the free PTE enzyme and with the two control enzyme‐nanoparticle host systems depicted in Figure 2a. The first control nanoparticle, pSiNP‐OH, lacking in the relatively thick surface oxide shell, was a nanoparticle type that we had previously shown to be able to encapsulate a nanoluciferase enzyme^[^ ^52^ ^]^ but in that study, the trapped enzyme showed reduced catalytic activity toward its substrate, relative to the free enzyme. The second control was OxpSiNP materials that had been chemically modified with N‐butyl terminal groups using a cyclic butyl‐azasilane (BAS) reagent (n‐n‐butyl‐aza‐2,2‐dimethoxysilacyclopentane),^[^ ^38^ ^]^ denoted as BAS‐OxpSiNP, prior to loading of the PTE enzyme. The BAS‐OxpSiNPs displayed a substantially greater surface hydrophobicity than the other two nanoparticle types, as discussed below. These controls aimed to investigate the impact of surface chemistry on the catalytic activity of the prepared enzyme@nanoparticle constructs, particularly regarding the influences of the surface oxide layer and of surface hydrophobicity. Detailed comparisons of the three nanoparticle hosts were conducted by water contact angle measurements and attenuated total reflectance Fourier‐transform infrared (ATR‐FTIR) spectroscopy (Figure S1, Supporting Information), nitrogen adsorption–desorption analysis (Figure S2 and Table S1, Supporting Information), thermogravimetric analysis (TGA, Figure S4, Supporting Information) and inductively coupled plasma mass spectrometry (ICP‐MS, Figure S5, Supporting Information).

Significant decreases in the BET surface area and pore volume were observed from the OxpSiNP and BAS‐OxpSiNP compared to the pSiNP‐OH samples. Because the first step in synthesis of either the OxpSiNP or the BAS‐OxpSiNP samples was thermal oxidation (at 700 °C) of a pSiNP‐OH preparation (Scheme 1), the nanostructure can be expected to have undergone significant volume expansion and other morphological changes as oxygen inserted into portions of the silicon skeleton to create the SiO_2_ shell. The observed decreases in surface area and in pore volume are thus attributed to a filling or blocking of the smaller pores in the nanostructure by this resulting thermal oxide shell.

Changes in average pore size that were observed upon formation of either of the OxpSiNP or the BAS‐OxpSiNP samples are consistent with the above interpretation; while the total pore volume and total surface area decreased, the average pore size of the samples increased relative to the pSiNP‐OH starting material (Figure S2a and Table S1, Supporting Information). Similar observations have been made previously in thermal oxidation studies of porous silicon particles—a decrease in pore volume and in surface area but an increase in average pore diameter.^[^ ^32^ ^]^ Based on Barrett–Joyner–Halenda (BJH) analysis of the nitrogen adsorption isotherms, the mean pore size increased from 13 ± 1 nm in pSiNP‐OH to 19 ± 2 nm in the OxpSiNP samples. The average pore size of the BAS‐OxpSiNP samples was larger still, though the difference in average pore size between the OxpSiNP and the BAS‐OxpSiNP samples was within the experimental error of the measurements. The pore size distribution data from the BJH analysis (Figure S2b–d, Supporting Information) reveal a broad distribution of pore sizes in the pSiNP‐OH samples. The corresponding curves for the OxpSiNP and the BAS‐OxpSiNP samples also show broad distributions of pore diameters, but the distribution curves show that the relative number of pores of the smallest diameters is substantially lower in these samples, consistent with a model in which oxidation closes off the smaller pores in the distribution. Oxide growth also induces strain in the porous material, and prior studies have indicated that strain can cause an overall enlargement of the pSi skeleton,^[^ ^32^ ^]^ which might also contribute to the observed increase in average pore diameter. We note that grafting of the butyl‐azasilane (BAS) reagent to the OxpSiNPs, which generated the BAS‐OxpSiNP formulations, resulted in no significant changes in measured average pore diameter compared to the parent OxpSiNPs (Figure S1b–d and Table S1, Supporting Information).

Infrared spectral measurements (Figure S1a, Supporting Information) confirmed the nature of the surface chemistry of the nanoparticles. The pSiNP‐OH nanoparticle preparations were formed under relatively mild oxidizing conditions by room‐temperature ultrasonic fracture of freshly etched porous silicon in water. The surface of freshly etched material is covered with surface Si─H (silicon hydride) species.^[^ ^57^ ^]^ Thus the ATR‐FTIR spectra of the pSiNP‐OH samples contained features assigned to residual unreacted Si─H surface species in the form of weak vibrational signatures appearing in the 2080–2160 cm^−1^ region of the spectrum. A strong Si─O (oxide) vibration in the 1000–1150 cm^−1^ region of the spectrum was indicative of significant surface oxidation. The hydrophilic nature of this oxide was confirmed by the presence of a broad O─H stretching band centered at ≈3300 cm^−1^ and a weak band at 1640 cm^−1^, assigned to the H─O─H scissors mode of adsorbed water. The data are consistent with water contact angle measurements performed on flat porous silicon films (still attached to the Si wafer substrate) that had been processed using the same chemistry (Figure S1b, Supporting Information).

The OxpSiNPs were prepared from pSiNP‐OH materials by thermal treatment at 700 °C in air (Scheme 1). The ATR‐FTIR spectra indicated complete elimination of the Si─H species and a prominent Si─O band in the 1000–1150 cm^−1^ region (Figure S1a, Supporting Information). The BAS‐OxpSiNP samples, prepared from OxpSiNPs by direct reaction with the cyclic silane, also displayed a strong Si‐O stretching band. In addition, characteristic bands for C─H bending and stretching modes of the grafted butyl groups, at 1480 and 2960 cm^−1^, were evident in BAS‐OxpSiNPs (Figure S1a, Supporting Information). When the same BAS chemistry was applied to flat porous silicon layers (still attached to the Si substrate), water contact angle measurements (Figure S1b, Supporting Information) confirmed a substantially more hydrophobic surface (water contact angle of 63°, compared to 15° for the parent OxpSi layer). TGA measurements performed on the BAS‐OxpSiNPs indicated that the nanoparticles contained 6.2 ± 0.3 wt% grafted silane species (Figure S4, Supporting Information). The OxpSiNP and the BAS‐OxpSiNP samples were substantially more stable toward dissolution in aqueous solution compared to the pSiNP‐OH samples. All three sample types were incubated in aqueous pH 7.4 buffer solutions for 24 h, the nanoparticles were removed, and the supernatants were assayed for dissolved silicon by ICP‐MS. The data showed >5‐fold lower concentration of dissolved silicon for either the OxpSiNP or the BAS‐OxpSiNP samples compared to pSiNP‐OH (Figure S5, Supporting Information).

The encapsulation of the enzyme PTE L7ep3a in pSiNP‐OH followed a previously published procedure,^[^ ^58^ ^]^ involving the treatment of pSiNP‐OH materials with aqueous HEPES buffer (at pH 7.4) in the presence of the enzyme to simultaneously induce mild oxidation of the pSiNP‐OH skeleton and trap the enzyme within the nanostructure. The enzyme‐loaded pSiNP‐OH samples displayed mean hydrodynamic diameters of 338 ± 43 nm and a zeta potential of −25.0 ± 0.6 mV in a pH 7.4 HEPES buffer solution (Figure S6a and Table S2, Supporting Information), hereafter denoted as PTE@pSiNP‐OH. PTE L7ep3a was loaded into the BAS‐OxpSiNP samples following a similar protocol to that described above for loading of PTE L7ep3a into OxpSiNPs. The PTE‐loaded BAS‐OxpSiNP samples are referred to here as PTE@BAS‐OxpSiNP. The PTE@BAS‐OxpSiNP samples exhibited significant aggregation upon enzyme loading; the mean hydrodynamic diameter increased from 307 ± 10 nm to 756 ± 32 nm. This suggests that electrostatic attractions between the net negative charge of the enzyme and the net positive charge of PTE@BAS‐OxpSiNPs at pH 7.4 may induce aggregation (Figure S6b and Table S2, Supporting Information). The zeta potential of BAS‐OxpSiNP displayed a net positive charge (+40.0 ± 3.1 mV), which is attributed to the presence of the secondary amine that is generated in the ring‐opening reaction that attaches the butyl azasilane group to the nanoparticle surface (see Figure 2a). The immobilization of enzyme (which is negatively charged at pH 7.4) to this surface led to a reduction in the value of the zeta potential, to +15.5 ± 1.8 mV (Figure S6c and Table S2, Supporting Information). The mass loading of the PTE L7ep3a enzyme in the pSiNP‐OH and BAS‐OxpSiNP materials was calculated to be 5.3 ± 1.2% and 4.0 ± 0.4%, respectively.

Next, the kinetic parameters associated with the catalytic hydrolysis of DMNP by the enzyme@nanoparticle constructs were compared. The hydrolysis of DMNP resulted in the formation of 4‐nitrophenol (Figure 2b), which exhibits a yellow color (λ_max_ = 400 nm) in a neutral buffer solution, facilitating the study of enzyme kinetics by spectrophotometry. Figure 2c presents a series of plots depicting the initial rates of conversion of DMNP to 4‐nitrophenol as a function of the initial concentration of DMNP, fitted using the Michaelis–Menten equation (Equation 1). The trace for PTE@OxpSiNP is compared with the two PTE@nanoparticle controls and with free PTE enzyme. For these experiments, the total concentration of PTE enzyme (whether free or bound in a nanoparticle) was held constant. The kinetic parameters derived from these measurements are presented in Table S3 (Supporting Information), and the determined values of *k_cat_* are compared in Figure 2d.

Remarkably, under identical assay conditions, PTE@OxpSiNPs displayed a roughly two‐fold enhancement in *k_cat_* relative to free PTE. In contrast, the PTE@pSiNP‐OH construct showed 4.5 times lower *k_cat_* relative to the free enzyme. The value of *k_cat_* represents the turnover number or the number of substrate molecules that one enzyme can convert to the product per unit of time.^[^ ^59^ ^]^ Interestingly, no significant difference was observed between the *k_cat_* values of PTE@BAS‐OxpSiNPs and free PTE L7ep‐3a, suggesting that the observed enhancement of enzyme activity upon immobilization in OxpSiNPs could be offset by an increase in surface hydrophobicity in the host. The PTE@BAS‐OxpSiNPs also displayed the smallest value of *k_cat_/K_m_* (Table S3, Supporting Information), which is a measure of how effectively the enzyme captures the substrate for catalytic turnover.^[^ ^59^ ^]^ Taken together, the kinetic data indicate that enzyme performance is highly dependent on the local environment introduced by the nanostructure, and that the OxpSiNP host provides the most favorable local environment for catalysis.

To understand the mechanistic reason for the compromised activity observed in the PTE enzyme when immobilized in BAS‐OxpSiNPs, it is helpful to consider the effect of the local environment in the pores of the nanoparticle, where catalysis occurs, on catalytic performance. Studies have shown that adsorption onto hydrophobic surfaces can negatively impact enzyme orientation and dynamics,^[^ ^60^, ^61^ ^]^ potentially resulting in a compromised hydrolysis rate for the PTE@BAS‐OxpSiNP construct. Additionally, previous studies have established that phosphate esters with leaving groups having a pK_a_ less than 7.5 undergo hydrolysis rapidly in the presence of wild‐type PTE, and the rate is limited by the product dissociation step.^[^ ^41^, ^62^ ^]^ This is of relevance here, since the product 4‐nitrophenol has a pKa of 7.15, suggesting that its reaction is primarily influenced by product dissociation rather than by hydrolysis. Using this interpretation, the net positive charge of PTE@BAS‐OxpSiNPs (Figure S6 and Table S2, Supporting Information) may impede the removal of the hydrolyzed product 4‐nitrophenol (which is in its deprotonated form under the pH 7.4 assay conditions) through electrostatic interactions. Furthermore, the hydrophobic surface microenvironment of BAS‐OxpSiNPs could influence the rate of reaction by changing the availability of water in the local vicinity of the trapped enzyme under the assay conditions.

While surface chemistry is likely important, nanoparticle size may also play a role in the observed reduction in enzyme activity for the PTE@BAS‐OxpSiNPs. As noted above, the PTE@BAS‐OxpSiNPs were ≈2‐fold larger than the other nanoparticles in this study due to aggregation effects. Previous studies have demonstrated that substantial changes in particle size are required to significantly impact the catalytic activity of immobilized enzymes. For example, Li et al.^[^ ^43^ ^]^ reported that increasing the size of a MOF enzyme host from 1 to 2 µm did not lead to a substantial reduction in catalytic activity, and Breger et al.^[^ ^42^ ^]^ showed that a ten‐fold increase in particle size resulted in only a 50% reduction in *k_cat_*.

Both the pSiNP‐OH and the OxpSiNP hosts showed a similar degree of hydrophilicity despite their dramatic differences in turnover rates, so the level of hydration within the nanopores can only be part of the story. The pSiNP‐OH samples were prepared by treating the porous Si material in aqueous ethanolic media at room temperature. This low‐temperature oxide readily dissolves in aqueous solutions to generate silicic acid,^[^ ^63^ ^]^ and the condensation of silicic acid within the pores could result in blocking of the pores that would limit substrate transport to the caged enzyme. In contrast, the OxpSiNPs were prepared from the pSiNP‐OH samples via thermal treatment in air at 700 °C. At this high temperature, more of the Si and Si─H core of pSiNP‐OH was converted to SiO_2_ in the nanoparticles, and this high‐temperature surface oxide in the Si─SiO_2_ core‐shell structure is substantially less susceptible to aqueous dissolution compared to the pSiNP‐OH material, therefore minimizing pore blocking due to the condensation of excess silicic acid.

For the OxpSiNP host, it is difficult to identify the predominant factor that led to the enhanced activity of the trapped enzyme relative to the free enzyme. Improvement in enzyme‐substrate association is not a likely explanation, as the *K_m_* value for the PTE@OxpSiNP construct was larger compared to free PTE, indicating a lower affinity of the enzyme for the substrate molecules when immobilized in the OxpSiNP host. We hypothesize that the enhanced enzyme activity in the PTE@OxpSiNP construct is due to the localized microenvironment in the OxpSiNPs, where the electrostatic repulsions between the negative pore walls and the negatively charged nitrophenoxide product enhance its expulsion from the pores of the nanoparticle. Such surface charge effects have been shown to influence the transport of negatively charged proteins within oxidized porous silicon.^[^ ^56^ ^]^ Although it is unclear what precisely is responsible for the enhanced catalytic activity, the key conclusion that can be drawn here is that OxpSiNPs provided an optimal host for the PTE L7ep3a enzyme for hydrolysis of OP compounds.

### 2.3 Effect of Mass Loading of Enzyme in the PTE@OxpSiNP Construct on Activity

Having established the optimized host for enzyme immobilization as OxpSiNPs, we next assessed the impact of the mass loading of enzymes in the pores of this nanoparticle type. Three levels of enzyme loading were obtained by using different concentrations of enzyme and a fixed amount of OxpSiNPs (1 mg mL^−1^) in the loading procedure, resulting in enzyme content ranging from 5.7 to 15.4% by mass (Table S3, Supporting Information). These samples are designated as low (L), medium (M), and high (H), i.e., PTE@OxpSiNPs_(L)_, PTE@OxpSiNPs_(M)_, and PTE@OxpSiNPs_(H)_ to reflect the relative density of enzyme in each sample type. The estimation of the mass loading of enzyme immobilized in the OxpSiNPs under each condition was determined from mass and spectral measurements as described in Table S3 (Supporting Information). Using this information and the pore volume (cm^3^ g^−1^) determined from the nitrogen adsorption measurements, the loading density, or mass of enzyme per available open pore volume in the samples was determined to be 72.3, 132.5, and 219.3 mg cm^−3^ for the PTE@OxpSiNPs_(L)_, PTE@OxpSiNPs_(M)_ and PTE@OxpSiNPs_(H)_ samples, respectively. The actual values may deviate from these calculated values due to an uneven nanoparticle size distribution or partial aggregation of nanoparticles. Figure 2e presents kinetics plots of DMNP hydrolysis using PTE@OxpSiNPs with the different mass loadings of enzyme. A decrease in *k_cat_* was observed as the enzyme mass loading increased (Figure 2f). This inverse relationship between catalytic activity of PTE@OxpSiNPs and enzyme mass loading is attributed to blocking of enzyme active sites as the enzyme molecules became more tightly packed into the pores, restricting enzyme‐substrate interactions.^[^ ^64^ ^]^ Thus lower enzyme mass loadings should result in reduced crowding within the pores, allowing for greater conformational flexibility and more accessibility to the enzyme active site, enhancing catalytic activity. The superior activity observed for the lower enzyme loading suggests that additional optimization of enzyme mass loading in PTE@OxpSiNPs could result in further increases in activity. However, limitations in accurately measuring immobilized protein concentrations constrained further exploration. The formulation with the lowest mass loading, PTE@OxpSiNPs_(L)_, was chosen for the subsequent studies discussed below due to its optimal catalytic activity for DMNP hydrolysis among the formulations tested.

### 2.4 Durability of the PTE@OxpSiNP Construct in Harsh Environments and for Repeated Use

**Figure 3:**
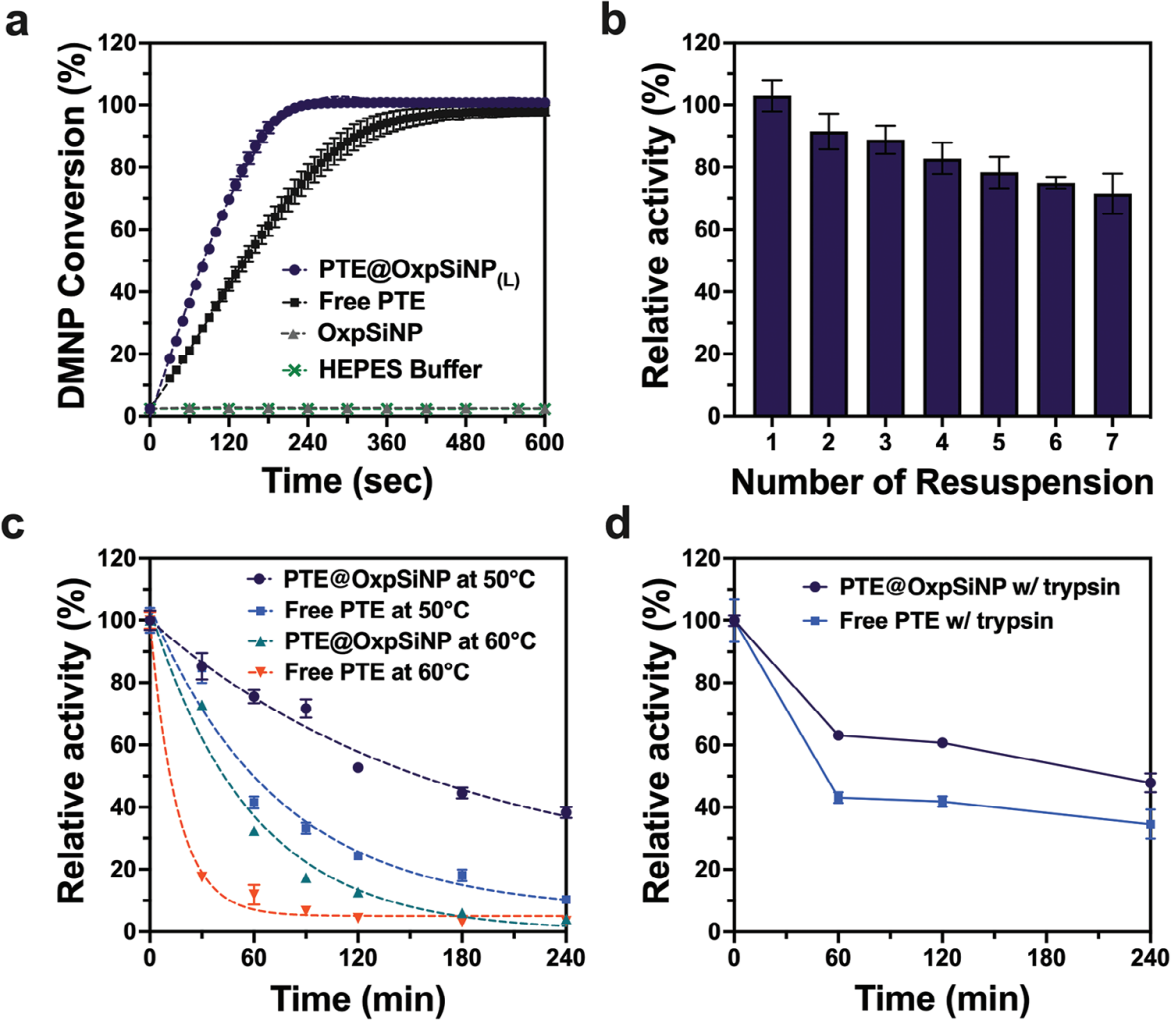
Durability of the PTE@OxpSiNP construct. (a) Percent DMNP conversion as a function of time by PTE@OxpSiNP (purple), free PTE (black), OxpSiNP (grey), and background reaction in pure buffer (green). The PTE@OxpSiNP and free PTE experiments contained an identical amount of PTE enzyme. (b) Activity assessment of the PTE@OxpSiNP construct for DMNP hydrolysis after repeated isolation and resuspension of the nanoparticles, comparing initial rates to the control measurement (fresh PTE@OxpSiNP without any washing steps). (c) Relative activity of PTE@OxpSiNP and free PTE as a function of incubation time at either 50 °C or 60 °C. The heated samples were allowed to cool to room temperature for 10 min before running the activity assays. Relative activity was quantified as the initial rate of reaction for DMNP conversion. Dashed lines are fits to a single‐exponential decay. (d) Relative activity of PTE@OxpSiNP and free PTE as a function of incubation time with 2 mg mL^−1^ trypsin at room temperature. All assays conducted at room temperature using a consistent effective PTE concentration of 1 µg mL^−1^ (27.4 nm) with 1 mm DMNP in HEPES buffer (50 mm, pH 7.4 containing 0.1 mm CoCl_2_). Error bars indicate standard deviations from three independent measurements and preparations.

To evaluate the potential of the PTE@OxpSiNP construct to detoxify OP compounds under real‐world conditions, we subjected the construct to various challenges. We first evaluated the conversion of DMNP to 4‐nitrophenol at pH 7.4 and room temperature to establish a baseline (**Figure** **3**a). Comparing PTE@OxpSiNPs to free PTE under the same conditions (and using the same mass of enzyme), the PTE@OxpSiNPs achieved 100% conversion of DMNP within 3 min, whereas free PTE required 5–6 min to achieve the same level of conversion. This outcome aligns with the predicted performance based on the kinetics measurements discussed above (Figure 2). Control experiments involving the incubation of DMNP with empty OxpSiNPs or simple HEPES buffer at pH 7.4 demonstrated no detectable activity toward DMNP hydrolysis.

We next tested the sturdiness of the OxpSiNP cage in trapping the PTE enzyme, by repeatedly dispersing a PTE@OxpSiNP sample into buffer and then isolating it by centrifugation, discarding the supernatant each time. The purpose of these experiments was to assess if there was substantial leaching of the immobilized enzyme from the host material. To examine this, 1 mg of the PTE‐loaded nanoparticles were dispersed into1 mL of HEPES buffer (50 mm, pH 7.4 with 0.1 mm CoCl_2_). The sample was then centrifuged and the supernatant (which potentially contained leached enzymes) was discarded. The nanoparticles were resuspended by brief sonication into a fresh buffer solution. A small aliquot of this PTE@OxpSiNP suspension was then assayed for enzyme activity. The volume of buffer used in each successive resuspension step was adjusted to account for the fractional loss of sample to the aliquots, such that the concentration of nanoparticles in each successive resuspension was identical. The nanoparticles retained ≈72% activity after seven successive washes, relative to a control PTE@OxpSiNP sample that was not subjected to the above washing steps (Figure 3b). We attribute this loss in observed activity to enzyme leaching from the particles, or to incomplete recovery of particles adhered to the walls of the centrifuge tubes during the washing steps. Notably, the PTE@OxpSiNP samples were still able to achieve ≈100% DMNP conversion within 5 min after the seventh wash (Figure S7a, Supporting Information). To assess reusability of a single PTE@OxpSiNP sample, we conducted ten consecutive DMNP neutralization cycles using the same batch of PTE@OxpSiNPs (200 µg). The nanoparticles were incubated in a 10 mm DMNP solution in HEPES buffer for 10 min, separated from the soluble 4‐nitrophenol hydrolysis product by centrifugation, and then redispersed in a fresh solution of 10 mm DMNP in HEPES buffer for subsequent assay. There was no significant reduction in DMNP conversion observed through the 10 cycles of reuse (Figure S7b, Supporting Information). These experiments demonstrated that repeated exposure to DMNP along with the reaction byproducts did not impair the biological activity of the immobilized enzyme.

We next evaluated the ability of the OxpSiNP host to protect the enzyme under more aggressive conditions. The PTE variant L7ep3a is known to have broad stability in the pH range 5–9, with slight loss in activity observed in the range from 4.5 to 5.^[^ ^50^ ^]^ Additionally, the dissolution of porous silicon nanoparticles is suppressed under acidic conditions.^[^ ^65^, ^66^ ^]^ This study therefore focused on assessing the protective effect of the OxpSiNP host on PTE Lep3a when subjected to high temperatures and proteolytic degradation conditions. The initial DMNP turnover rates of PTE@OxpSiNPs and free PTE were measured post‐exposure to 50 °C and 60 °C for defined durations (Figure 3c). These are temperatures well above skin temperature but ones that might be encountered on the surface of operating machinery or a surface exposed to bright sunlight. The initial DMNP turnover rates of both PTE@OxpSiNPs and free enzyme at RT (25 ± 1 °C) served as a 100% activity benchmark for each formulation, respectively. Not surprisingly, the free enzyme exhibited a rapid decline in activity, with estimated half‐lives (t_1/2_) of 51 and 11 min at 50 and 60 °C, respectively (Figure S8, Supporting Information). Notably, PTE@OxpSiNPs displayed t_1/2_ values of 117 and 41 min at 50 and 60 °C, indicating a 2.3‐ and 3.7‐fold increase in stability compared to free enzyme (Figure S9, Supporting Information).

To assess the ability of the OxpSiNP cage to shield an entrapped enzyme from proteolytic degradation, PTE@OxpSiNPs, and free enzyme were incubated with the proteolytic enzyme trypsin (2 mg mL^−1^) at room temperature. As depicted in Figure 3d, PTE@OxpSiNPs exhibited slightly improved resistance to trypsin digestion after a 4‐h incubation, retaining 48% of their original activity, compared to 35% for free enzyme (Figure S10, Supporting Information). This modest protection from trypsin digestion indicates that entrapped enzyme in the OxpSiNPs is still accessible to the relatively smaller (23 kDa) porcine trypsin protein. Overall, this data suggests that immobilizing PTE L7ep3a using OxpSiNPs confers some degree of resistance to proteolytic degradation compared to free enzyme.

Finally, to assess temporal storage stability of the PTE@OxpSiNP samples, we subjected them to pelletization by centrifugation and removal of the buffer solution, storing them at 4 °C for a period of 7 days. Stability was evaluated by measuring the DMNP conversion over time using the assays discussed above. Measurement of percent DMNP conversion as a function of time for the PTE@OxpSiNP samples stored at 4 °C for 7 days showed no significant difference compared to the as‐prepared PTE@OxpSiNP samples (Figure S11a, Supporting Information). In addition, we evaluated lyophilization as a method for longer‐term storage of the enzyme@nanoparticle constructs. PTE@OxpSiNP samples lyophilized with trehalose were stored at various temperatures (25, 4, −20, and −80 °C) for up to 8 weeks. The optimal storage condition for the lyophilized powders was determined to be 4 °C; the lyophilized powders retained >75% activity after 8 weeks of storage at this temperature (Figure S11b, c, Supporting Information).

### 2.5 Degradation of VX by PTE@OxpSiNP on an Ex Vivo Rabbit Skin Model

Nerve agent VX presents a complex challenge for detoxification due to its persistence in the environment, its low volatility, and its notably high toxicity compared to other nerve agents (Acute Exposure Guideline Level 3, lethal: 0.096–0.62 mg min m^−3^ for VX versus 3.8–24.5 mg min m^−3^ for GB).^[^ ^67^ ^]^ The engineered enzyme variant PTE L7ep3a used in this study has shown significant enhancement in hydrolyzing V‐type (VX and VR) nerve agents compared to the wild‐type PTE.^[^ ^50^, ^51^ ^]^ Hence, VX serves as a pertinent target for evaluating the detoxification potential of the PTE@OxpSiNP construct against live nerve agents. To evaluate the ability of PTE@OxpSiNPs to counter VX poisoning through skin exposure, we formulated a gel composed of PTE@OxpSiNPs (0.2 wt %) and Carbopol 940, a crosslinked polyacrylic acid polymer, and applied it onto rabbit skin samples in a Franz cell diffusion apparatus (Figure S12, Supporting Information). The Franz diffusion apparatus simulated transdermal penetration of VX, where rabbit skin samples treated with the PTE@OxpSiNP‐formulated gel were placed between donor and acceptor chambers. A VX solution (5 µL, 1 mg µL^−1^) was introduced by topical application to the treated or untreated skin on the donor side of the chamber. The acceptor chamber contained tris‐buffered saline (TBS). Quantification of VX retention or penetration through rabbit skins was accomplished by extracting aliquots from the acceptor side of the chambers and then subjecting them to liquid chromatography tandem quadrupole mass spectrometry (LC‐MS/MS).

**Figure 4:**
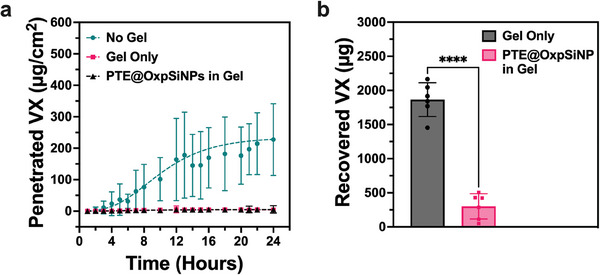
Detoxification of the nerve agent VX by a gel containing the PTE@OxpSiNP construct on an ex vivo skin model. (a) VX penetration, in micrograms of VX per cm^2^ of exposed rabbit skin over a 24‐h period in a Franz cell setup. Experiments were conducted with no treatment (No Gel, green), Carbopol gel alone (Gel Only, pink), and Carbopol gel containing 0.2 wt % PTE@OxpSiNPs (black) applied to the rabbit skins. Data are means ± SD (n = 5) (b) Quantification (by LC/MS/MS) of VX recovered from the rabbit skin samples treated with Carbopol gel (Gel Only) and Carbopol gel containing PTE@OxpSiNPs. Data are means ± SD (*n* = 6); *p* values were determined by unpaired *t*‐test, with ^****^ *p* < 0.0001 indicating significant difference.

**Figure** **4**a shows that the Carbopol gel was effective at preventing VX penetration through the rabbit skin over a 24‐h period, either with or without the PTE@OxpSiNPs, while VX penetrated the untreated rabbit skins. Analysis of the VX‐exposed gel after 24 h revealed at least a six‐fold reduction in the quantity of VX in the PTE@OxpSiNP‐containing gel relative to gel alone (Figure 4b). Although Carbopol gel itself acted as a physical barrier against VX skin penetration, its inability to detoxify VX exposed on the skin surface poses risks of secondary exposure. Here, only the PTE@OxpSiNP construct was tested on the ex vivo rabbit skin model due to its superior in vitro catalytic performance compared to free PTE. The results demonstrated that the gel did not deactivate the ability of the enzyme‐nanoparticle construct to turn over the VX substrate. Due to the nature of the experiments, the turnover rates and other kinetic parameters of the reaction were not determined.

It is important that any potential decontaminant agent in a topical gel be highly biocompatible. Despite being effective for catalytic hydrolysis of nerve agents, catalysts involving metal oxide nanoparticles like cerium oxide (CeO_2_) or metal‐organic frameworks (MOFs) have raised concerns regarding their dermal toxicity^[^ ^68^, ^69^ ^]^ and other safety issues, limiting their use in applications such as skin protectant gels. To assess the dermal safety of the PTE@OxpSiNPs, an in vitro sensitization assay (IVSA) and an Epiderm Skin Irritation Test (SIT) were performed using human‐derived keratinocytes. The IVSA quantified interleukin (IL‐18) released from keratinocytes as an indicator of an inflammatory skin response to an irritant. This assay showed negligible effect to PTE@OxpSiNP challenges administered in a range of 32–10 000 µg mL^−1^, similar to non‐irritant controls (Figure S13a and Table S4, Supporting Information). Similarly, an MTT assay coupled with SIT, which utilized 3D reconstructed human epidermis tissues, exhibited no significant decrease in tissue viability after treatment with PTE@OxpSiNPs at concentrations up to 10 mg mL^−1^ (Figure S13b, Supporting Information). Control experiments involving incubation of PTE@OxpSiNPs with the MTT assay reagents displayed no coloration, eliminating the possibility of false‐positive results that have been seen with some porous silicon samples.^[^ ^70^ ^]^ These results affirm the suitability of PTE@OxpSiNPs for use in biomedical countermeasures against organophosphonate nerve agents.

### 2.6 Evaluation of Neutralization of VX Toxicity in Human Blood: AChE Inhibition Studies

**Figure 5:**
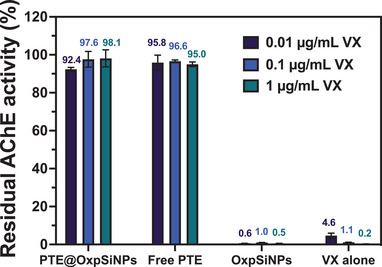
Results of AChE activity assay in human blood, evaluating effectiveness of PTE@OxpSiNPs in neutralizing VX. Data comparing acetylcholine esterase activity in human blood that was treated with solutions containing VX which had previously been treated with PTE@OxpSiNPs, with free PTE, with OxpSiNPs (blank nanoparticles containing no enzyme) or with nothing (VX alone). The effective enzyme concentration in PTE@OxpSiNPs was 10 µg mL^−1^ with a total particle concentration of 200 µg mL^−1^; the concentration of free PTE was 200 µg mL^−1^. Error bars represent the standard deviation of three independent measurements.

In a separate set of experiments, we performed in vitro AChE activity assays in human blood to provide a separate, functional readout of the efficacy of the PTE@OxpSiNPs in deactivating VX agent. The motivation for these experiments was two‐fold: first, to assess if unreacted VX remained at toxic levels, and second, to assess if any of the products of the catalytic reaction were neurotoxic—though prior work has shown that enzymatic action of PTE on VX generates non‐toxic hydrolysis products,^[^ ^50^, ^51^ ^]^ there is a possibility that the nanoparticle‐immobilized enzyme might generate a different set of products. Nerve agents and some of their hydrolysis products exert their toxic effects by irreversible inhibition of acetylcholinesterase (AChE), which halts the breakdown of the neurotransmitter acetylcholine, leading to neurological impairment and eventual death by asphyxiation.^[^ ^1^ ^]^ To perform the assay, neutral buffer solutions of VX were incubated with the PTE@OxpSiNPs for 1 h, followed by centrifugation to separate the supernatants. These supernatants were then aliquoted and mixed with a reaction mixture containing AChE in human blood solution and Ellman's reagent (5,5′‐dithio‐ bis‐[2‐nitrobenzoic acid], DNTB). Subsequently, the AChE activity was assayed by adding the substrate acetylthiocholine into the reaction mixture to quantify the breakdown of acetylthiocholine catalyzed by AChE through a colorimetric reaction (λ_max_ = 450 nm; see the Experimental Section for details). The data (**Figure** **5**) showed that PTE@OxpSiNPs were able to retain > 90% of AChE activity across all tested VX concentrations (0.01–1 µg mL^−1^), displaying the same capability to prevent AChE inhibition as observed with free enzyme; indeed, the effective concentration of enzyme in the nanoparticle experiments was 20‐fold lower than in the experiments that used free enzyme. Control experiments conducted without PTE L7ep3a (OxpSiNPs and VX alone) showed the expected diminished AChE activity. Combined with the results shown in Figure 4, these results highlight the potential of PTE@OxpSiNPs to act as effective neutralizers for chemical nerve agents.

## 3 Conclusion

This work demonstrated the successful immobilization of a phosphotriesterase enzyme, the PTE variant L7ep3a, into a partially oxidized porous Si‐based nanoparticle host (PTE@OxpSiNP). Mild oxidation of the porous Si material in aqueous ethanolic media at room temperature (pSiNP‐OH) was not sufficient to provide good performance and a secondary thermal oxidation step at 700 °C (OxpSiNP) was needed to enhance the stability and the performance of the final enzyme@nanoparticle construct. Once subjected to the high‐temperature thermal treatment, the OxpSiNPs could be loaded with the enzyme by simply soaking the particles in an aqueous buffer solution containing the enzyme at room temperature. The OxpSiNPs spontaneously concentrated the PTE enzyme into their interior (by ≈400‐fold relative to the free solution concentration) and the procedure restructured the silicon oxide such that PTE was effectively locked inside the OxpSiNP cage. A side‐by‐side comparison of the hydrolysis kinetics of the organophosphorus (OP) simulant DMNP by PTE@OxpSiNPs with PTE‐containing control constructs revealed the significant impact of the surface chemistry of the nanoparticle host on the catalytic activity of the enzyme‐immobilized construct. The results suggest that low packing density of enzyme, and the local hydration state within the pores, were important in achieving high catalytic activity. The PTE@OxpSiNP construct enabled a higher DMNP hydrolysis rate compared to other control PTE‐immobilized constructs and even outperformed the free enzyme. The optimized PTE@OxpSiNP construct afforded additional benefits including reusability and improved tolerance of the enzyme to elevated temperatures and to enzymatic proteolysis. The PTE@OxpSiNP construct remained catalytically active when formulated with a hydrogel‐based cream, and the functional cream effectively hydrolyzed the live nerve agent VX in an ex vivo rabbit skin model. The ability of the construct to detoxify VX was further validated with an in vitro AChE activity assay. The PTE@OxpSiNP particles showed a high dermal safety profile, with no observed irritation or decrease in viability of a human‐derived epidermis tissue model. While this study indicates that the porous silicon‐based nanoparticle cages impart remarkably improved performance to the enzyme, longer‐term stability and efficacy tests over a wider range of environmental conditions are needed to validate the suitability of this approach for real‐world decontamination applications.

## 4 Experimental Section

### Materials

N‐(2‐Hydroxyethyl)piperazine‐N′‐(2‐ethanesulfonic acid) (HEPES), Dimethyl p‐nitrophenyl phosphate (DMNP) were purchased from Sigma‐Aldrich. N‐n‐butyl‐aza‐2,2‐dimethoxysilacyclopentane (BAS) was purchased from Gelest. Fluorescein N‐hydroxysuccinimide (FAM‐NHS) was purchased from Lumiprobe. Trypsin (from porcine pancreas) was obtained as a lyophilized powder from Milipore Sigma (catalog number T4799). Other chemicals or reagents were of analytic grade and used without further purification. Porous silicon flakes (L634 formulation, Porosity: 80 ±  10%, Pore size: 10–20 nm, derived from highly boron‐doped p‐type wafers) were purchased from TruTag Technologies, inc.

### Preparation of pSiNP‐OH, OxpSiNP, and BAS‐OxpSiNP Materials

Porous silicon flakes obtained from the vendor were fractured by ultrasonication in 20% ethanolic solution in deionized water for ≈18 h at room temperature. The ultrasonic bath used in this procedure was not thermostated, and so the temperature of the bath after 18 h was somewhat higher than ambient. The brown suspension collected after sonication was centrifuged at 15 000 rpm (relative centrifugal force, or rcf = 16 000 g) for 10 min, and the bottom pellet was washed, sonicated, centrifuged two times each in absolute ethanol, and the supernatants were discarded to remove particles of sizes <100 nm. The resulting pellet was then re‐suspended and centrifuged at 2500 rpm (rcf = 400 g) for 5 min and the pellet was discarded to eliminate the micron‐sized and larger particles. The resulting pSiNP‐OH material remaining in the supernatant was stored in absolute ethanol for further use.

To prepare OxpSiNP material, the pSiNP‐OH samples were first dried at 80 °C in air for 30 min and placed in a ceramic boat which was then inserted into a preheated tube furnace at 700 °C in air for 45 min. After the thermal oxidation treatment, the ceramic boat containing the particles was moved to the edge of the tube furnace to cool for 2 min, and it was then removed from the furnace to allow cooling to room temperature. The obtained oxidized particles were again sonicated in a 20% solution of ethanol in deionized water for ≈18 h to fully disperse the thermally oxidized particles. The suspension collected after the sonication step was centrifuged at 2500 rpm (rcf = 400 g) for 5 min to eliminate the large particles and stored in absolute ethanol for further use.

To prepare BAS‐OxpSiNP material, the OxpSiNPs were washed, sonicated, and centrifuged two times each in tetrahydrofuran (THF) to remove residual protic solvents. OxpSiNPs (1 mg) were suspended in n‐n‐butyl‐aza‐2,2‐dimethoxysilacyclopentane (BAS) solution (10% v/v) in THF (1 mL) and the mixture was agitated for 1 h. The suspension collected after the reaction was washed, sonicated, and centrifuged two times each in absolute ethanol.

### Preparation of PTE@pSiNP‐OH Constructs

The pSiNP‐OH samples (2 mg mL^−1^) were suspended in HEPES buffer (50 mm, pH = 7.4, containing 0.1 mm CoCl_2_) with PTE (70 µg mL^−1^) at room temperature under stirring for 2 h. The PTE‐loaded nanoparticles were recovered by centrifugation at 15 000 rpm (rcf = 16 000 g) for 10 min, and then washed, sonicated, and centrifuged two times each in HEPES buffer (50 mm, pH = 7.4, containing 0.1 mm CoCl_2_) to remove loosely adsorbed PTE. The loading and washing solutions were collected for quantification of mass loading. The amount of enzyme loaded into the nanoparticles was calculated from the initial and final concentrations of the loading and washing solution (*n* = 3) using UV absorbance spectrophotometry at 280 nm.

### Preparation of PTE@OxpSiNP Constructs

The OxpSiNPs (2 mg mL^−1^) was suspended in HEPES buffer (50 mM, pH = 6.5, containing 0.1 mm CoCl_2_) with PTE (70 µg mL^−1^) at room temperature under stirring for 2 h. The PTE‐loaded nanoparticles were recovered by centrifugation at 16 000 g for 10 min, and then washed, sonicated, and centrifuged two times each in HEPES buffer (50 mm, pH = 6.5, containing 0.1 mm CoCl_2_) to remove loosely adsorbed PTE. The loading and washing solutions were collected for quantification of mass loading. The amount of enzyme loaded into the nanoparticles was calculated from the initial and final concentrations of the loading and washing solution (*n* = 3) using UV absorbance spectrophotometry at 280 nm.

### Preparation of PTE@BAS‐OxpSiNP Constructs

The BAS‐functionalized OxpSiNPs (2 mg mL^−1^) was suspended in HEPES buffer (50 mm, pH = 6.5, containing 0.1 mm CoCl_2_) with PTE (70 µg mL^−1^) at room temperature under stirring for 2 h. The PTE‐loaded nanoparticles were recovered by centrifugation at 16 000 g for 10 min, and then washed, sonicated, and centrifuged two times each in 50 mm HEPES buffer (pH = 7.4, containing 0.1 mm CoCl_2_) to remove loosely adsorbed PTE. The loading and washing solutions were collected for quantification of mass loading. The amount of enzyme loaded into the nanoparticles was calculated from the initial and final concentrations of the loading and washing solution (*n* = 3) using UV absorbance spectrophotometry at 280 nm.

### Preparation of Fluorescein‐Tagged Enzyme

Fluorescein N‐hydroxysuccinimide (FAM‐NHS) and PTE were suspended in HEPES buffer solution (50 mm, pH = 7.4, containing 0.1 mm CoCl_2_) and left in darkness at 4 °C under static condition (without stirring) for 24 h. The FAM‐tagged PTE was purified by passing the reaction mixture through a 30 kDa centrifugal filter (1200 g for 10 min), followed by buffer exchange with fresh HEPES (50 mm, pH = 7.4, containing 0.1 mm CoCl_2_). The purification and buffer exchange processes were repeated five times to ensure the washed solution displayed no visible color from the FAM dyes. The obtained FAM‐tagged PTE solution was stored in darkness at 4 °C.

### Scanning Electron Microscopy (SEM)

SEM images were obtained with a Zeiss Sigma 500 field‐emission scanning electron microscopy (FESEM). Prior to analysis, the samples were dispersed in ethanol, drop‐cast onto a piece of silicon wafer fragment, and mounted on 12 mm aluminum sample stubs. The samples were then sputter‐coated with iridium at 85 mA for 10 s using a Emitech K575X sputter coater.

### Nitrogen Adsorption/Desorption Analysis

Nitrogen adsorption isotherms were obtained on dry particles with a Micromeritics ASAP 2020 instrument. For each run, samples (10 mg) were placed in a ball‐shaped end glass analysis tube and degassed under vacuum for 5 h at 80 °C prior to measurement. The isotherms were then analyzed at a temperature of 77 K to determine the Brunauer–Emmet–Teller (BET) surface area using ASAP 2020 software.

### Thermogravimetric Analysis (TGA)

TGA data was obtained on TA Instruments Discovery SDT 650. ≈5 mg of sample was placed on a disposable sample pan and heated at 100 °C for 30 min to remove the residual solvent trapped within the samples. The sample was then heated to 800 °C in a pure nitrogen atmosphere at a rate of 10 °C min^−1^ to determine the mass loss.

### Dynamic Light Scattering (DLS) and Zeta Potential Measurements

For hydrodynamic size, the particles before and after the PTE loading (each 2 mg mL^−1^) were each dispersed in HEPES buffer (50 mm, pH = 7.4, 0.1 mm CoCl_2_). Thereafter, an aliquot (20 µL) of the particle solution was diluted by the same buffer solution to yield final samples (67 µg mL^−1^) for the measurements. The measurement was performed on a Malvern Zetasizer ZS90 DLS instrument using a polystyrene cuvette. For zeta potential, the particles before and after the PTE loading were dispersed in the HEPES buffer at a concentration of 67 µg mL^−1^ and measured with a folded capillary zeta potential cell (DTS1070). Both measurements were performed at 25 °C.

### Confocal Laser Scanning Microscopy (CLSM)

The loading and spatial distribution of the fluorophore‐tagged enzyme in and on the surface of the pSiNPs was characterized using a Leica TCS SPE confocal microscope. The sample was excited at *λ_ex_* = 488 nm and the fluorescence signal was collected in the wavelength range 490–550 nm. The spatial distribution of enzyme was visualized by a z‐stacking technique over a range of 25 µm.

### Determination of Reaction Kinetic Parameters for Free PTE and PTE@pSiNP Constructs

PTE kinetics assays were carried out in Thermo Fisher clear, flat‐bottom 96‐well microplates. For a typical run, DMNP solutions (50 µL) at a range of concentrations (125, 250, 500, 1000, 2000, and 3000 µm) were prepared and added horizontally into six wells of the plate. A stock solution of DMNP (100 mm) was first prepared in methanol and a serial dilution was then carried out using HEPES buffer (50 mm, pH = 7.4, 0.1 mm CoCl_2_) to prepare the DMNP solutions. Thereafter, solutions containing free PTE or different PTE@pSiNP constructs (50 µL, with an effective concentration of PTE at 2 µg mL^−1^) were added to another horizontal row of the plate corresponding to each DMNP concentration. Using a multichannel pipette, the solutions containing free PTE or PTE@pSiNP constructs were added to the DMNP solutions. Each well contains: DMNP (62.5–1500 µm) and PTE (1 µg mL^−1^ or 27.4 nm) dispersed in HEPES buffer (50 mm, pH 7.4, containing 0.1 mm CoCl_2_). Formation of *p*‐nitrophenol, the DMNP hydrolysis product, was monitored by change in absorbance at λ_abs_ = 400 nm in 10‐s increments over 10 min at 25 °C. Absorbance values were converted to concentration by employing a *p*‐nitrophenol standard curve in HEPES buffer (50 mm, pH 7.4 containing 0.1 mm CoCl_2_). All experiments were performed in triplicate. The catalytic activity was measured as initial rate for each DMNP concentration, taken from the linear range where DMNP conversion was <10%. The obtained initial rates were plotted against DMNP concentration and the kinetic parameters were calculated using the Michaelis–Menten kinetics model given by the equation shown below using GraphPad Prism software.

(1) $$V=\frac{V_{max}\left[S\right]}{K_{m}+\left[S\right]}=\frac{E_{t}k_{cat}\left[S\right]}{K_{m}+\left[S\right]}$$

where *[S]* was substrate DMNP concentration (µm), *V_max_* was the maximum rate attained at infinite concentration of substrate, and *K_m_* was the Michaelis–Menten constant. The term *V_max_* can be further expressed in terms of *E_t_* and *k_cat_*, which were the total enzyme concentration used in the kinetics study and the turnover number, respectively.

### Temperature and Protease Degradation Assays

For the thermal stability tests, free PTE (10 µg mL^−1^) or PTE@OxpSiNP constructs (1 mg mL^−1^) in HEPES buffer (50 mm, pH 7.4 containing 0.1 mm CoCl_2_) were incubated at 50 and 60 °C, respectively. For proteolysis tests, free PTE or PTE@OxpSiNP constructs (with the same PTE concentration of 50 µg mL^−1^) were incubated with trypsin solution (2 mg mL^−1^) in HEPES buffer (50 mm, pH 7.4 with 0.1 mm CoCl_2_) at room temperature. In both assays, aliquots of free PTE and caged PTE constructs (20 µL) were taken at different time intervals and diluted with HEPES buffer to yield final samples (with an effective concentration of PTE at 2 µg mL^−1^). Thereafter, the increase in absorbance at λ_max_ = 400 nm was monitored over 10 min at 25 ± 1 °C every 10 s after adding aliquots of free PTE or PTE@OxpSiNP constructs (50 µL) to DMNP solutions (50 µL, 2 mm) in HEPES buffer. The final concentration of PTE was 1 µg mL^−1^ (27.4 nm) and the DMNP concentration was 1 mm. The enzyme activity for each condition was determined from the initial rate of DMNP hydrolysis and was compared to a room temperature (RT, 25 ± 1 °C) control experiment.

### VX Dermal Challenge on Ex Vivo Rabbit Skin Model

CAUTION: Due to the acute toxicity associated with VX, all experiments involving VX were performed by qualified personnel in certified chemical fume hoods equipped with an advanced filtration system that protects the user and the environment at the Combat Capabilities Development Command (CCDC) Chemical Biological Center (Edgewood, MD, USA) according to all Federal, State, and International guidelines. The rabbit skin samples were mounted in an automated transdermal diffusion cell sampling system (Logan Instruments, Somerset, NJ) with the stratum corneum facing the donor chamber, exposing a surface area of 1.77 cm^2^ for VX penetration. The acceptor chamber held 1X tris‐buffered saline (TBS, 12 mL) with a magnetic stir bar, maintained at 32 °C. Gels (1 g) with and without the PTE@OxpSiNP constructs were uniformly applied to the skin surface, followed by the application of neat VX (5 µL, purity >95%). Sampling (1 mL) was conducted from the acceptor chamber at predetermined intervals (0.08, 1, 2, 3, 4, 5, 6, 7, 8, 10, 12, 13, 14, 15, 16, 18, 20, 21, 22, and 24 h) followed by an addition of fresh TBS (1 mL) to maintain a constant volume.

Quantification of VX at each time interval was performed using LC‐MS/MS, employing an Agilent Technologies 1100 series liquid chromatograph coupled to a 6410 triple‐quadrupole mass spectrometer. Samples (1 µL) were diluted with methanol (49 µL) containing internal standard ^2^H_5_‐VX (200 ng mL^−1^), and subsequent injections (1 µL) were made with a constant flow rate of 1 mL min^−1^ through an Agilent Zorbax Eclipse XDB‐C_18_ (4.6 × 150 mm, 5 µm) analytical HPLC column with a C_18_ precolumn filter. The separation was achieved with an isocratic mobile phase consisting of 60% organic phase (0.1% formic acid in methanol) and 40% aqueous phase (0.1% formic acid in deionized water) over a 3‐min run, with target compound and internal standard peaks eluted at 1.4 min. The LC–MS/MS system operated in positive polarity mode, with an electrospray ionization source, gas temperature set at 350 °C, a flow rate of 10 L min^−1^, nebulizer pressure at 35 psi, and a capillary voltage of 4000 V. Multiple reaction monitoring (MRM) mode was employed. For the target analyte, the MRM program monitored one transition for quantitation (mass‐to‐charge ratio [m/z] 268 > 128) and one for confirmation (m/z 268 > 86), while for the internal standard ^2^H_5_‐VX, one transition was monitored for quantitation (m/z 273 > 128). A calibration curve across a concentration range of 0.5–10 000 ng mL^−1^ of VX was pre‐established with an isotopically labeled internal standard concentration of 200 ng mL^−1^ in methanol. After sampling, the gel on the skin surface was removed, and VX retained in the gel was extracted by dissolving it in isopropyl alcohol. Residual VX on the skin surface was removed by immersing the skin samples in isopropyl alcohol. The resulting solutions were combined and analyzed for VX concentration using the same protocol.

### Hydrolysis of VX and Acetylcholinesterase (AChE) Inhibition Studies

PTE@OxpSiNP constructs (200 µg mL^−1^) were incubated in three different concentrations of VX (0.01, 0.1 and 1 µg mL^−1^) for 1 h at 37 °C. The mixtures were then centrifuged at 20 817 g for 2 min to separate the materials from the hydrolyzed VX present in the supernatants. Whole human blood (100 µL, male, pooled; BioIVT) was pipetted into diH_2_O (20 mL) to lyse the red blood cells, followed by the addition of 10X phosphate buffer (2 mL) to stop the reaction. To a 96‐well microplate, lysed human blood samples (180 µL), the supernatants (20 µL) from the VX/PTE@OxpSiNPs mixtures, and Ellman's reagent (25 µL, 5,5′‐dithio‐bis‐[2‐nitrobenzoic acid], DNTB) were mixed and incubated at 37 °C for 10 min. Thereafter, AChE substrate reagent (25 µL) was added to the mixture and the AChE activity assay was performed by monitoring the increase in absorbance at λ_max_ = 450 nm over 25 min (45 readings) at room temperature. The same experiments were also performed based on free PTE under the same assay condition. A positive control experiment measuring AChE activity in the assay buffer was defined as 100% AChE activity.

### Dermal Safety Assays

The in vitro sensitization assay (IVSA), the EpiDerm Skin Irritation Test (SIT), and MTT (3‐(4,5‐dimethylthiazol‐2‐yl)‐2,5‐diphenyltetrazolium bromide) cell metabolic assay coupled with SIT were performed by MB Research Laboratories; experimental details are provided in the Supporting Information.

## Conflict of Interest

M.J.S. is a scientific founder (SF), a member of the Board of Directors (BOD), Advisory Board (AB), Scientific Advisory Board (SAB), acts as a paid consultant (PC), or has an equity interest (EI) in a number of companies.

## Supporting information



Supporting Information

## Data Availability

The data that support the findings of this study are available in the supplementary material of this article.
