# Supplementary material for: Engineering a Mesoporous Silicon Nanoparticle Cage to Enhance Performance of a Phosphotriesterase Enzyme for Degradation of VX Nerve Agent
Source: Adv Sci (Weinh). 2024 Nov 4;11(48):2409535. doi: 10.1002/advs.202409535 (PMC11672247; doi:10.1002/advs.202409535)
Supplement: Supplementary file 1 — Supporting Information [file ADVS-11-2409535-s001.pdf]

ADVANCED SCIENCE
Open Access

Supporting Information

for *Adv. Sci.*, DOI 10.1002/advs.202409535

Engineering a Mesoporous Silicon Nanoparticle Cage to Enhance Performance of a Phosphotriesterase Enzyme for Degradation of VX Nerve Agent

*Yi-Sheng Lu, Eduardo Reynoso Moreno, Yubin Huang, Ruhan Fan, Ashley T. Tucker, Linnzi K. Wright, Ronald A. Evans, Brooke M. Ahern, Donald E. Owens, Stephen A. Chappell, Dale J. Christensen, John Dresios and Michael J. Sailor**

Supporting Information

for

"Engineering a Mesoporous Silicon Nanoparticle Cage to Enhance Performance of a Phosphotriesterase Enzyme for Degradation of VX Nerve Agent"

*Yi-Sheng Lu, Eduardo Reynoso Moreno, Yubin Huang, Ruhan Fan, Ashley T. Tucker, Linnzi K. Wright, Ronald A. Evans, Brooke M. Ahern, Donald E. Owens, Stephen A. Chappell, Dale J. Christensen, John Dresios, and Michael J. Sailor**

Additional experimental procedures

Recombinant PTE L7ep3a-His$_6$ Expression and Purification

Materials

BL21 (DE3) competent E. coli strain (NEB C2527)
pET-20b(+) bacterial expression plasmid (Novagen 69739-3)
Luria Broth Base (Invitrogen 12795-084)
Terrific Broth (Fisher BP2468-500)
Carbenicillin (GoldBio C-103-25)
IPTG (isopropylthio-β-galactoside) (GoldBio I2481C25)
Ni-NTA Agarose (Qiagen)
Poly-Prep Chromatography Column (Bio-Rad 731-1550)
InstantBlue Coomassie Protein Stain (Abcam ab119211)
Anti-His Monoclonal Antibody (ThermoFisher Scientific 4E3D10H2/E3)

Buffers

PTE Lysis Buffer: 50mM HEPES pH 7.4, 1mM EDTA, 5% Glycerol, 1% CHAPS, 1mM β-mercaptoethanol, protease inhibitor cocktail (Roche), 100mg lysozyme (Sigma L6876)
PTE Wash Buffer: 50mM HEPES pH 7.4, 300mM NaCl, 5% Glycerol, 20mM Imidazole
PTE Elution Buffer: 50mM HEPES pH 7.4, 100mM NaCl, 5% Glycerol, 250mM Imidazole
PTE Dialysis Buffer: 50mM HEPES pH 7.4, 100mM NaCl
PTE Reaction Buffer: 50mM HEPES pH 7.4, 0.1mM $CoCl_2$

Plasmid Construction. Plasmid pET20b(+) harboring the PTE variant L7ep-3a (Bigley, 2005) was kindly provided by Dr. Frank Raushel. Primers were used to encode a C-terminal 6XHis tag on the PTE gene for affinity chromatography purification.

Expression and Purification. The PTE-6XHis plasmid was transformed into competent BL21 DE3 expression cells per manufacturer's instructions. The following day, a fresh 5mL LB culture was inoculated with a single transformed colony containing 100ug/mL Carbenicillin and 0.5% glucose and grown overnight at 37°C shaking at 200 RPM. The overnight culture was used to inoculate a fresh 2-liter culture containing 1-liter TB media and 100ug/mL Carbenicillin and allowed to grow at 37°C shaking at 200 RPM for 16 hours or until absorbance (O.D.600) reached 0.8. PTE-6XHis expression was induced by adding 1mM IPTG and 0.1mM $CoCl_2$ and grown for an additional 30 hours at 16°C shaking at 200 RPM. The bacteria were then pelleted by centrifugation at 4,500 RCF for 10 minutes. The pellet was resuspended in 50mM HEPES pH7.4 buffer to wash off residual LB media and then centrifuged at 4,500 RCF for 10 minutes. The pellet was then gently resuspended in 30mL PTE Lysis Buffer (50mM HEPES pH7.4, 1mM EDTA, 5% Glycerol, 1% CHAPS, 1mM beta-mercaptoethanol, 1X protease inhibitor cocktail, 100mg lysozyme). The resuspended pellet was then stored at -80°C until purification.

To purify recombinant PTE-6XHis protein, the bacterial suspension was thawed in a 37°C water bath for 1 hour. Lysate was transferred to a pre-chilled 50mL glass beaker with 300mM NaCl and 20mM Imidazole. To reduce lysate viscosity, the sample was sonicated on ice for 15 minutes (5 seconds "ON", 45 seconds "OFF") at 60% amplitude. Lysate was centrifuged at 20,000 RCF for 20 minutes to pellet the insoluble fraction. Next, the supernatant was transferred to a new 50mL conical tube and 500uL of Ni-NTA Agarose resin pre-equilibrated with 50mM HEPES pH7.4 buffer was added. Lysate was mixed with the Ni-NTA resin by a rotating platform for 1 hour at room temperature. Next, the lysate-Ni-NTA resin mixture was applied to a disposable 10mL Poly-Prep Chromatography column. Unbound protein was wash for a total of 20 column volumes using PTE Wash Buffer (50mM HEPES pH7.4, 300mM NaCl, 5% Glycerol, 20mM Imidazole). Bound protein was eluted by adding 3mL of PTE Elution Buffer (50mM HEPES pH7.4, 100mM NaCl, 5% Glycerol, 250mM Imidazole) in 500uL fractions. The eluted protein fractions were analyzed by SDS-PAGE gel and stained by Coomassie (Abcam ab119211) to confirm PTE-6XHis molecular weight and purity. Anti-His Western blotting was also performed to further verify PTE-6XHis protein. Fractions containing PTE-6XHis were then dialyzed overnight in 1-liter PTE Dialysis Buffer (50mM HEPES pH7.4, 100mM NaCl).

Reference:

Bigley, A. N.; Mabanglo, M. F.; Harvey, S. P.; Raushel, F. M. Variants of Phosphotriesterase for the Enhanced Detoxification of the Chemical Warfare Agent VR. Biochemistry 2015, 54, 5502-5512

In-Vitro Sensitization Assay (IVSA)

EpiDerm™ tissues (Lot No. 37887, Kit K) from MatTek Corporation were stored refrigerated (2 to 8°C) before undergoing equilibration at 37 ± 1°C, 5 ± 1% CO2, with MatTek assay medium for one hour. Following this, the tissues were transferred to fresh medium and topically exposed to the samples.

For the experiment, PTE@OxpSiNP and control samples were treated in duplicate on 24 EpiDermTM tissues. Each concentration of PTE@OxpSiNP samples (25 µL, 0.0032 – 10 mg mL-1), positive control (lactic acid, 30 µL, 2% w/v), and negative control (dinitrochlorobenzene, 30 µL, 5% w/v) were applied on two EpiDerm™ tissues in six-well plates. Two tissues treated with deionized water served as a baseline for calculating the IL-18 stimulation index, while two tissues were left untreated. After a 24-hour incubation at 37 ± 1 °C, 5 ± 1% CO2, assay media were collected and assayed for IL-18 cytokine concentration. Samples were analyzed immediately or stored at -20 °C until the samples can be tested concurrently.

Subsequently, the samples (150 µL) were added to the IL-18 Enzyme-Linked Immuno-Sorbent Assay (ELISA) kit plate (Medical and Biological Laboratories Co., LTD) and incubated for 1 hour at room temperature. After four washes with provided wash solution, anti-human IL-18 antibody conjugated with peroxidase (100 µL) was added to each well, followed by another incubation and four washes. A substrate reagent containing chromogen (100 µL) was added, incubated for 30 minutes, and the reaction was terminated with H_2SO_4 solution (100 µL, 0.5 M). The optical density (O.D.) at 450 nm was measured, and values were converted to IL-18 concentrations based on a predetermined calibration curve, established by IL-18 reference standards. The stimulation index (SI) was calculated as the ratio of secreted IL-18 concentration to each tested sample divided by that of the mean deionized water control experiments. MB Research Laboratories provided the testing services and data processing.

EpiDerm™ Skin Irritation Test (SIT)

EpiDerm™ tissues were equilibrated at 37 ± 1°C, 5 ± 1% CO_2, with assay medium (MatTek) for one hour, and were transferred to fresh medium for an additional overnight equilibrium for 18 ± 3 hours. The equilibration medium was replaced with fresh medium before dosing.

Each treatment including the PTE@OxpSiNP samples and controls was conducted in triplicate. On each EpiDerm™ tissue, 30 µL of both 5 and 10 mg mL-1 PTE@OxpSiNP samples were applied, followed by the placement of a nylon mesh to ensure uniform distribution. Simultaneously, a negative control (30 µl of DPBS) and a positive control (30 µl of 5% SDS solution) were assessed, employing a similar mesh for material distribution. The exposure duration for both the test article and controls was set at 60 minutes. After dosing and incubation, the tissues were rinsed with DPBS, gently blotted to remove excess substance, dried, and transferred to fresh medium. These rinsed EpiDerm™ tissues were then returned to the incubator for 24 ± 2 hours. Medium replacement occurred at 24 ± 2 hours, followed by an additional 18 ± 2 hours in the incubator.

Subsequently, each EpiDerm™ tissue was transferred to a 24-well plate containing 300 µl of MTT solution (1 mg/ml MTT in DMEM) and placed back into the incubator for an MTT incubation period of 3 hours ± 10 minutes. Following this incubation, each tissue was rinsed with DPBS and treated with 2.0 ml of extractant solution (isopropanol) per well for at least two hours at room temperature with shaking. Subsequently, two 200-µl aliquots of the extracted MTT formazan were transferred to a 96-well plate and measured at 570 nm using a plate reader (µQuant Plate Reader, Bio-Tek Instruments, Winooski, VT). MB Research Laboratories provided the testing services and data processing.

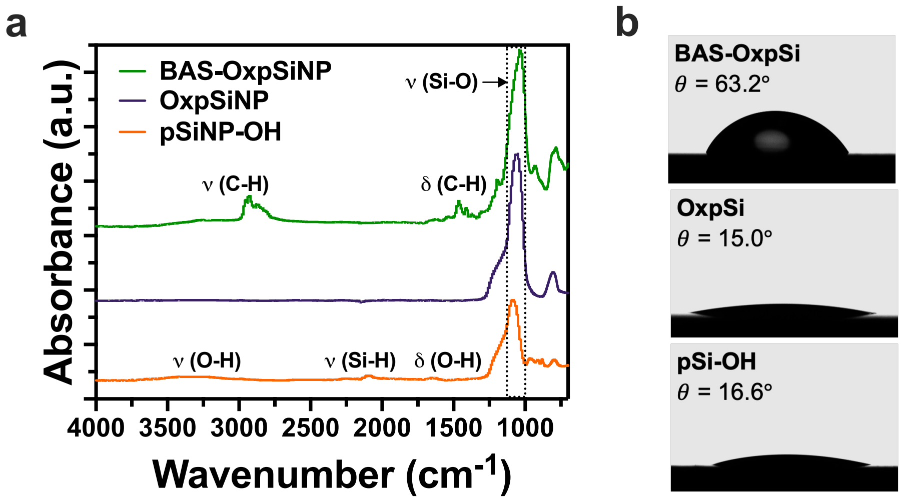

Figure S1. (a) Attenuated total reflectance Fourier-transform infrared (ATR-FTIR) spectra of pSiNP-OH, OxpSiNP and BAS-OxpSiNP samples, acquired from dry powders. (b) Water contact angles measured on porous films (still attached to their crystalline Si wafer substrate) of pSi-OH, OxpSi, and BAS-OxpSi. The presented values represent the average of triplicate measurements (S.D. ± 5°). Identical treatments involving thermal oxidation and N-butyl-azasilane grafting were applied to both the films and nanoparticles.

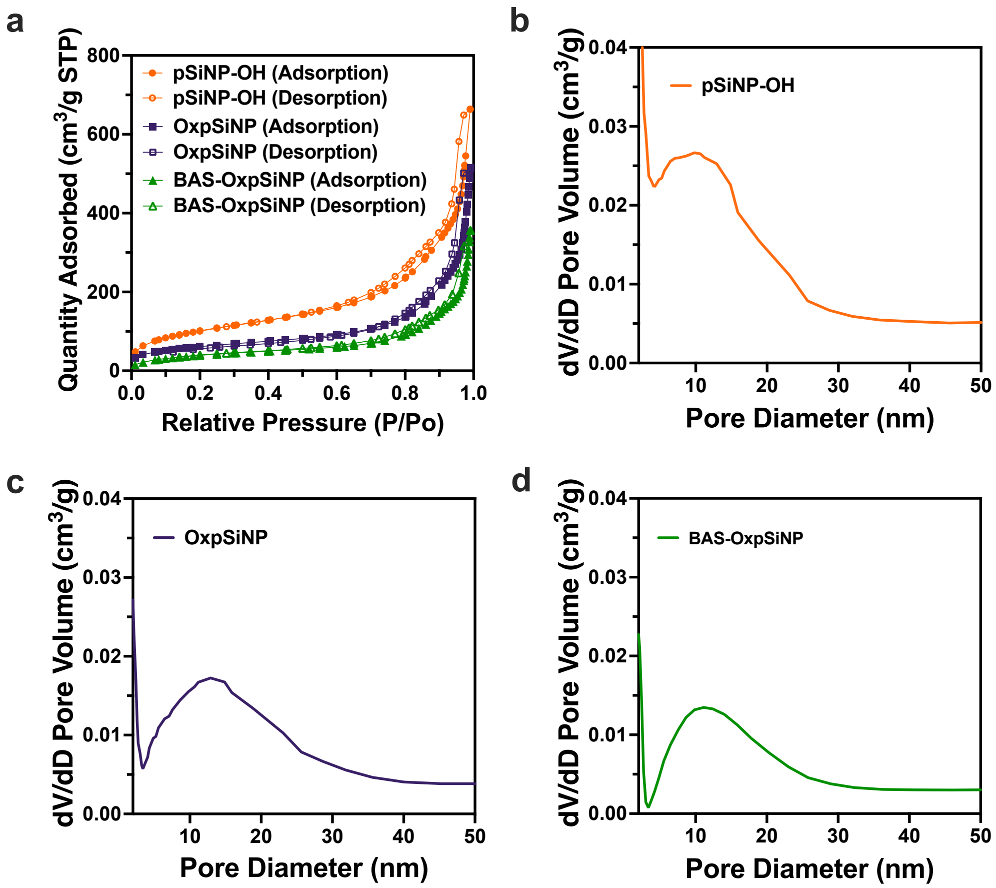

Figure S2. Cryogenic nitrogen adsorption/desorption isotherms (a) and pore size distribution for pSiNP-OH (b), OxpSiNP (c), and BAS-OxpSiNP (d). The surface area, pore volume and pore size were determined using BET (Brunnauer–Emmett–Teller) and BJH (Barrett–Joyner–Halenda) methods, and the values are given in Table S1.

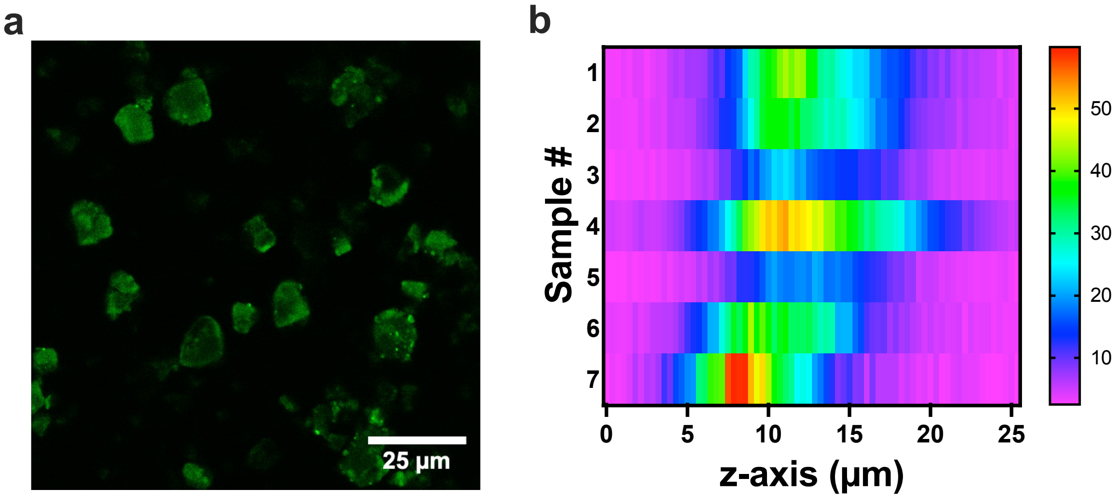

Figure S3. (a) Confocal microscope image showing the enzyme PTE L7ep3a immobilized within thermally oxidized porous silicon microparticles. Prior to the immobilization process, the enzyme was labeled with fluorescein N-hydroxysuccinimide (FAM-NHS), emitting a green fluorescence. (b) A heat map showing the intensity of fluorescence from the FAM-labeled enzyme within the pseudo one-dimensional pores of a sampling of 7 thermally oxidized pSi microparticles, spatially resolved in the z-direction. The fluorescence of the FAM-labeled enzyme was imaged using z-stack measurements spanning a 25 μm range, and the fluorescence intensity was quantified using the ImageJ software package (NIH).

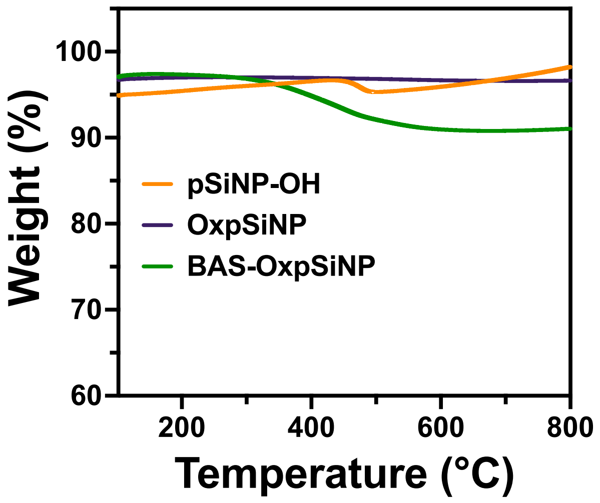

Figure S4. Thermogravimetric analysis (TGA) showing the weight changes of pSiNP-OH, OxpSiNP, and BAS-OxpSiNP samples, heated at a temperature ramp of 10 °C/min under an atmosphere of nitrogen. Triplicate runs were performed on each sample.

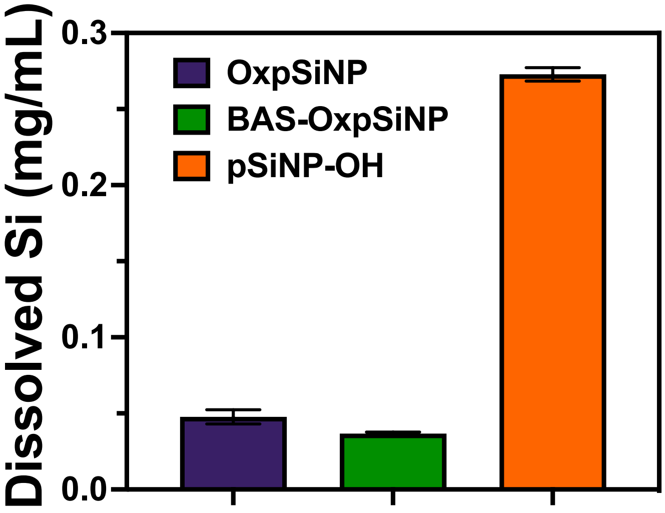

Figure S5. Dissolved silicon concentration measurements of OxpSiNP, BAS-OxpSiNP, and pSiNP-OH samples, as indicated, following a 24-hour incubation in HEPES buffer (50 mM, pH 7.4 with 0.1 mM $CoCl_2$) at room temperature. The quantification of dissolved Si was performed using the supernatants from each sample, after centrifugation to remove suspended nanoparticles, and analyzed via inductively coupled plasma mass spectrometry (ICP-MS). The $CoCl_2$ was added to these experiments to be consistent with the enzymatic assay experiments, where cobalt ion is a cofactor to enable enzyme activity.

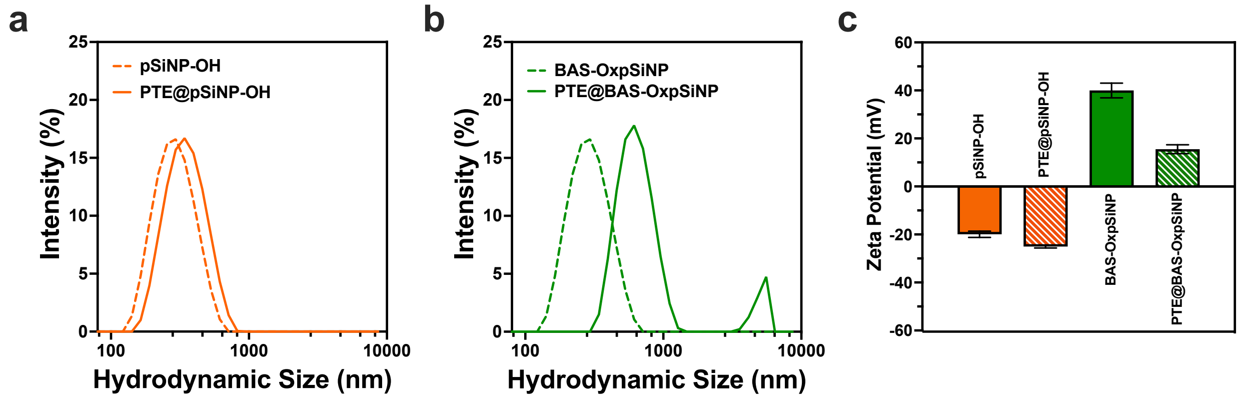

Figure S6. Hydrodynamic size distributions of (a) pSiNP-OH and (b) BAS-OxpSiNP samples in HEPES buffer (50mM, pH7.4 containing 0.1 mM $CoCl_2$), before and after the immobilization of the enzyme PTE L7ep3a. (c) Zeta potential measurements comparing both pSiNP-OH and BAS-OxpSiNP before and after the immobilization of PTE L7ep3a. Error bars represent the standard deviation of three independently prepared samples.

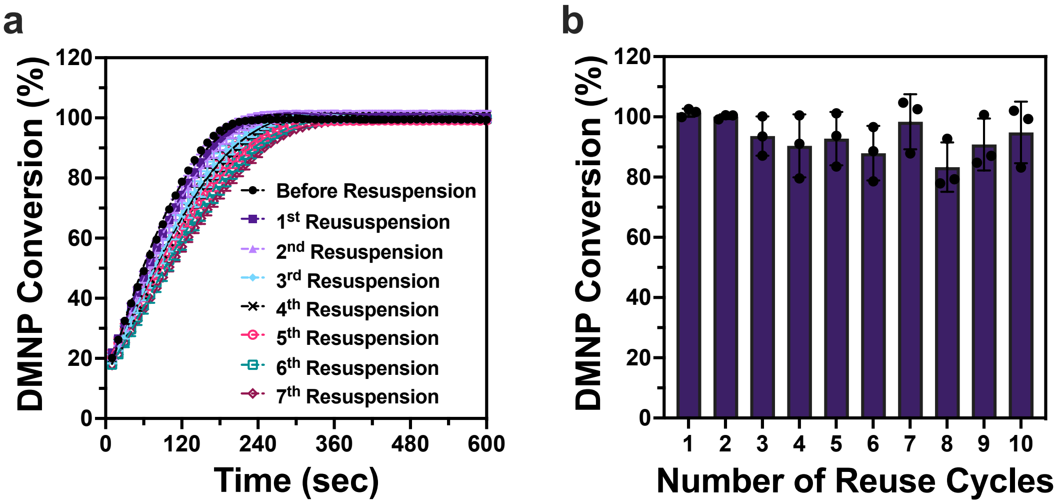

Figure S7. Test for leaching of enzyme from the PTE@OxpSiNP construct, and for the reusability of a PTE@OxpSiNP sample for repeated decontamination, quantified by measuring DMNP hydrolysis. (a) Percent hydrolytic conversion of DMNP as a function of time. Traces shown represent assay of 8 aliquots of a PTE@OxpSiNP catalyst suspension, taken from a sample that had been subjected to 7 sequential washing steps. Washing was achieved by suspension of the nanoparticles in HEPES buffer (50mM, pH 7.4 with 0.1 mM $CoCl_2$) and then isolating them from the buffer by centrifugation. The supernatant was discarded to remove any free enzyme that had potentially leached from the nanoparticles, the nanoparticles were resuspended in fresh buffer, and then a 20 µL aliquot was extracted from the PTE@OxpSiNP solution after each resuspension cycle and diluted with the HEPES buffer to achieve an effective enzyme concentration of 1 µg mL^{-1} (27.4 nM) along with 1 mM DMNP and the sample was assayed for enzyme activity by optical absorbance measurements for 10 minutes. Initial DMNP turnover rates of PTE@OxpSiNP samples following each resuspension cycle were compared to a control experiment without any resuspension. (b) Assay of a single PTE@OxpSiNP sample (200 µg mL^{-1}) through 10 cycles of full DMNP neutralization. The nanoparticles were incubated in a 10 mM DMNP solution in HEPES buffer for 10 minutes, the 4-nitrophenol hydrolysis product was quantified, and then the nanoparticles were separated from the 4-nitrophenol in the supernatant by centrifugation. The nanoparticles were then redispersed in a fresh buffer solution containing 10 mM DMNP for subsequent assay. Error bars represent the standard deviation derived from three independent measurements of three separately prepared samples. Measurements and manipulations were all performed at room temperature.

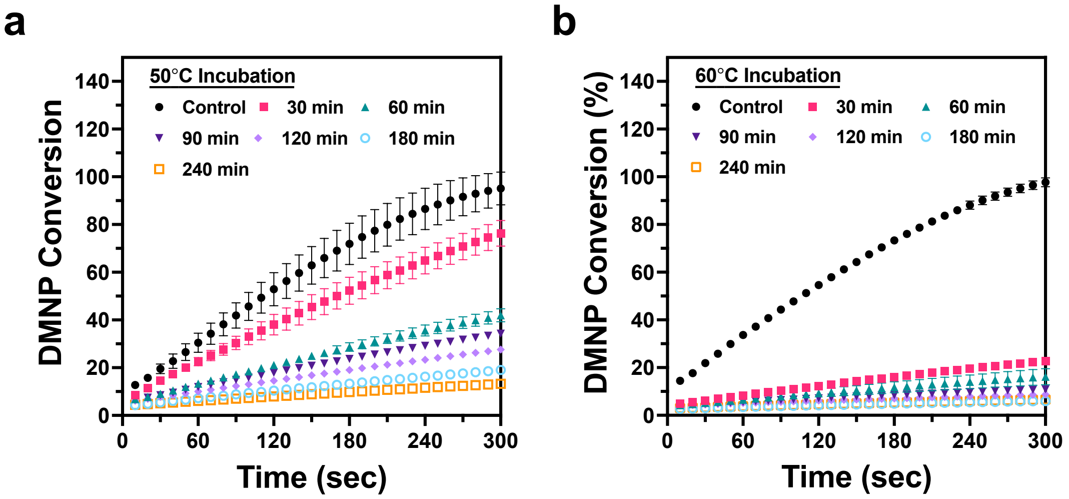

Figure S8. DMNP conversion over time by free PTE L7ep3a after exposure to (a) 50 °C and (b) 60 °C for 30 to 240 minutes in HEPES buffer (50mM, pH 7.4 with 0.1 mM $CoCl_2$). The samples were allowed to cool to room temperature for 10 minutes prior to addition of the DMNP substrate. The measurements were performed at room temperature using the same effective concentration of PTE at 1 μg mL^{-1} (27.4 nM) along with 1 mM DMNP. Error bars represent the standard deviation derived from three independent measurements of three independently prepared samples.

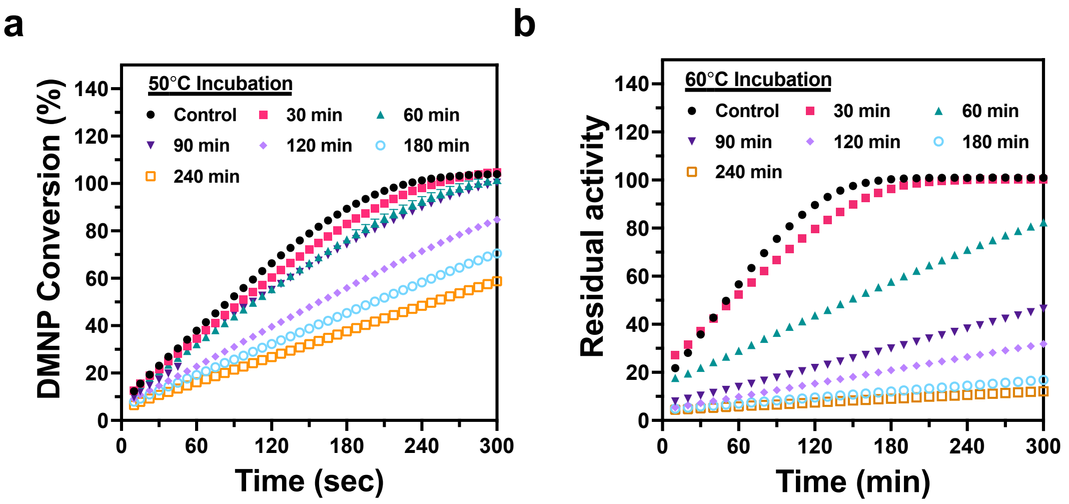

Figure S9. DMNP conversion over time by PTE@OxpSiNP samples after exposure to (a) 50 °C, and (b) 60 °C for 30 – 240 minutes in HEPES buffer (50mM, pH 7.4 with 0.1 mM $CoCl_2$). The samples were allowed to cool to room temperature for 10 minutes prior to addition of the DMNP substrate. The measurements were performed at room temperature using the same effective concentration of PTE at 1 µg mL^{-1} (27.4 nM) along with 1 mM DMNP. Error bars represent the standard deviation derived from three independent measurements of three independently prepared samples.

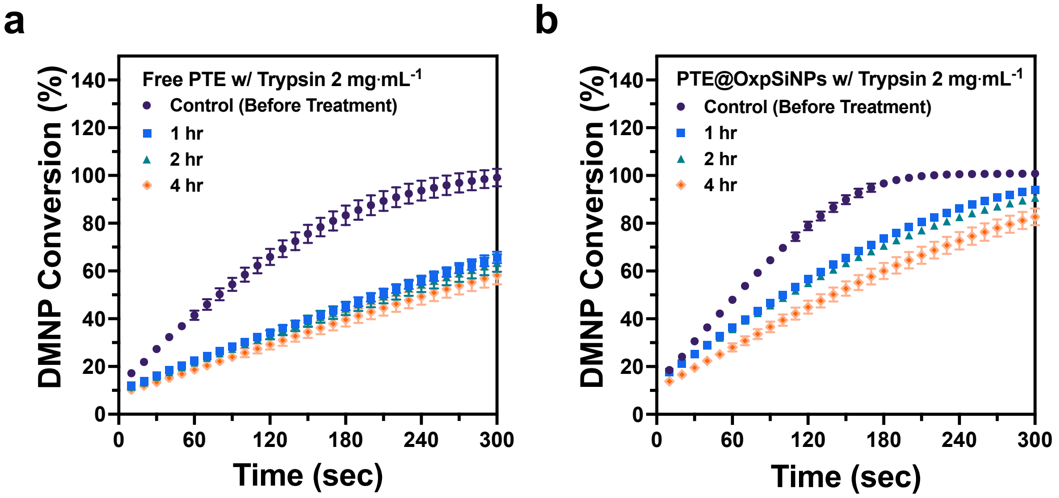

Figure S10. Conversion of DMNP as a function of time for (a) free PTE and (b) PTE@OxpSiNP after exposure to 2 mg mL^{-1} trypsin for 60 to 240 minutes (as indicated) in HEPES buffer (50mM, pH 7.4 with 0.1 mM $CoCl_2$). The assays were performed at room temperature using the same effective concentration of PTE at 1 μg mL^{-1} (27.4 nM) along with 1 mM DMNP. Error bars represent the standard deviation derived from three independent measurements of three independently prepared samples.

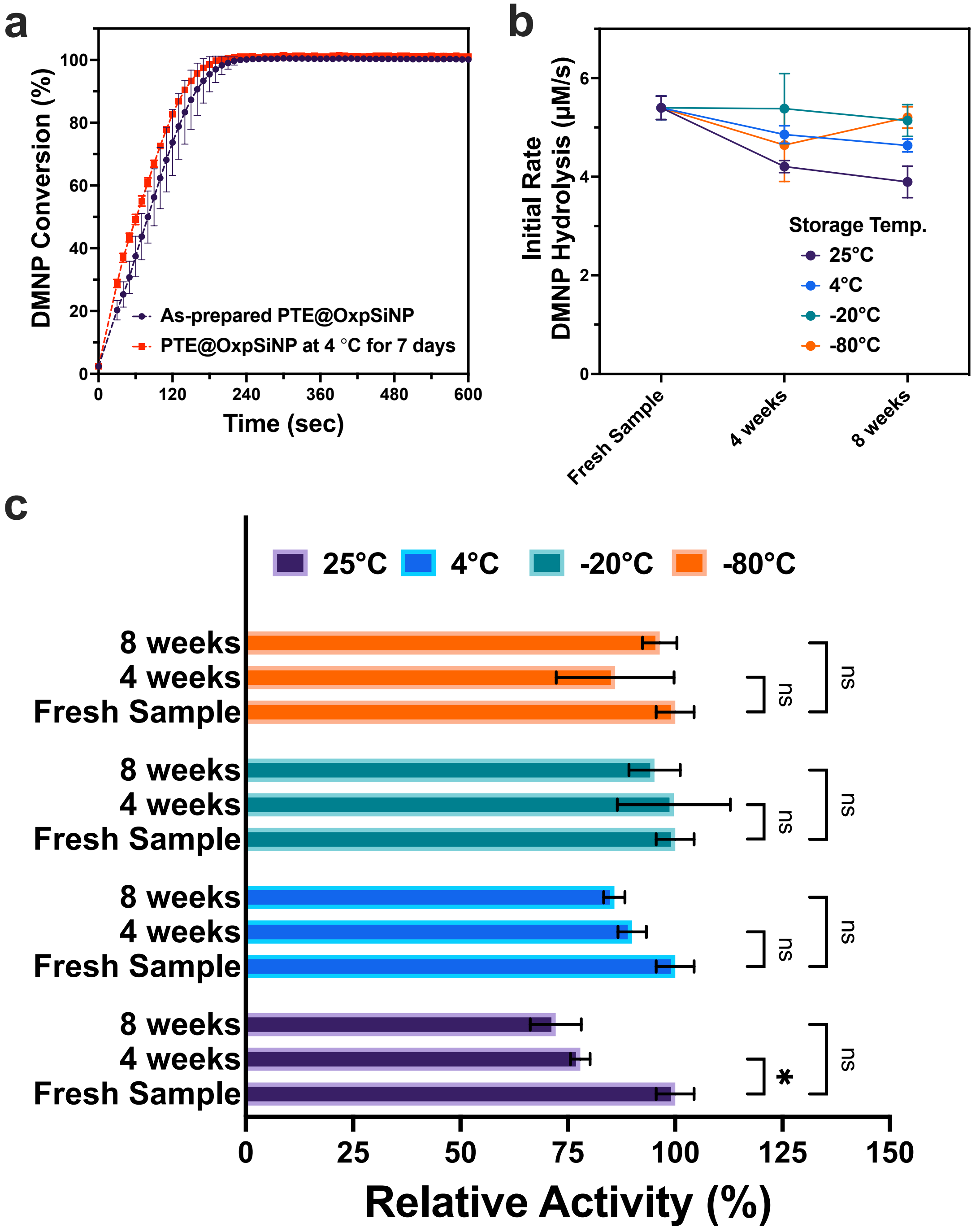

Figure S11. Storage stability of the PTE@OxpSiNP construct. (a) Conversion of DMNP as a function of time for as-prepared PTE@OxpSiNPs and after storage at 4 °C for a period of 7 days in HEPES buffer (50mM, pH 7.4 with 0.1 mM $CoCl_2$). (b) Initial rate, and (c) relative activity for DMNP hydrolysis using lyophilized PTE@OxpSiNPs stored at different temperatures (25, 4, -20 and -80 °C) for 4 and 8 weeks. The assays were performed at room temperature using the same effective concentration of PTE at 1 μg mL^{-1} (27.4 nM) along with 1 mM DMNP. Error bars represent the standard deviation derived from three independent measurements. Tukey's multiple comparisons test was used for the data (*$p < 0.05$; n.s., not significant).

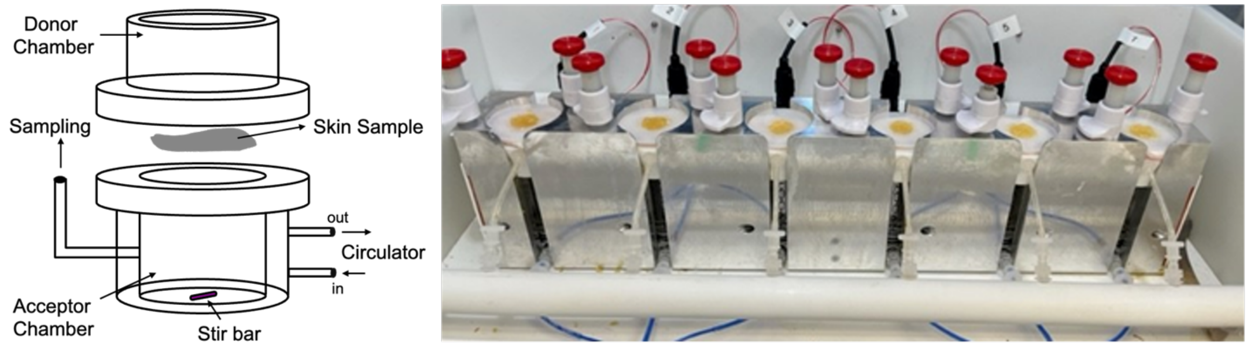

Figure S12. Schematic of a Franz diffusion cell (left): A layer of PTE@OxpSiNP-formulated gel (1 g, containing 0.2 wt % PTE@OxpSiNPs) was topically applied onto a rabbit skin sample. The skin sample was placed between the donor and acceptor chambers. Liquid VX was introduced by topical application to the treated or untreated skin on the donor chamber side. Photograph (right) showing the Franz cell setup conducting simultaneous tests on 6 rabbit skin samples in parallel. The photograph was taken before the introduction of VX.

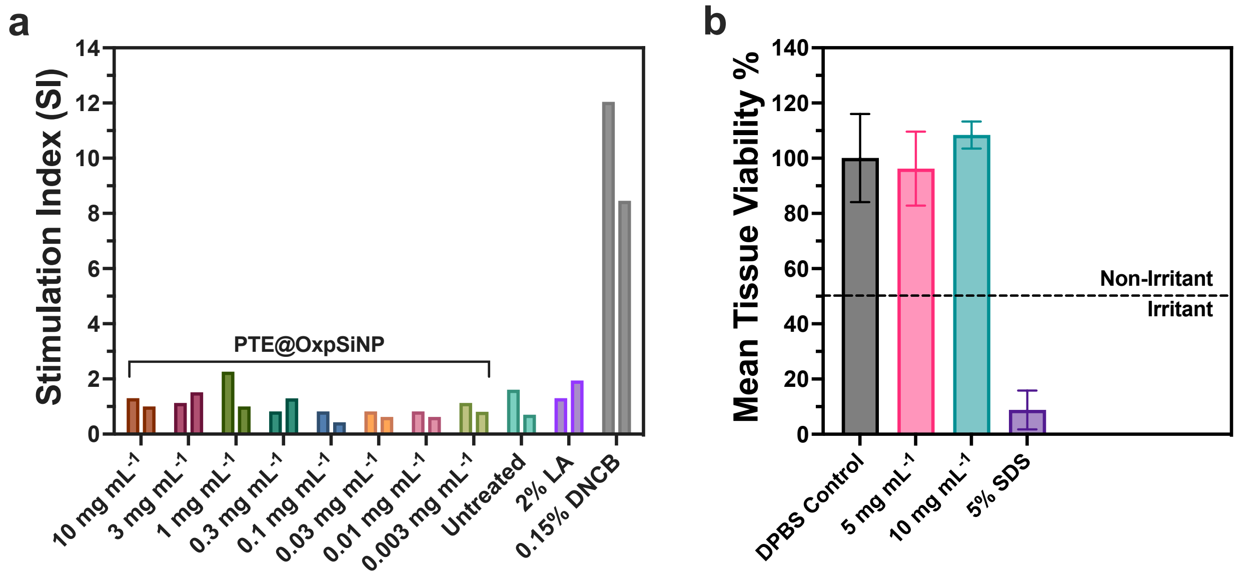

Figure S13 (a) *In-vitro* sensitization assay (IVSA) quantifying the keratinocyte-secreted interleukin-18 (IL-18) in response to treatment with PTE@OxpSiNP (0.003 – 10 mg mL^{-1}), compared to controls: 2% lactic acid (LA) as a negative control, and 0.15 % 1-chloro-2,4-dinitrobenzene (DNCB) as the positive control. IL-18 sensitization is presented as the fold change in the secreted IL-18 concentration relative to the H_2O control value. Individual data (from duplicate experiments) are shown corresponding to each treatment condition. (b) MTT assay quantifying mean tissue viability after a 42-hour incubation with PTE@OxpSiNP (5 and 10 mg mL^{-1}) at 37 °C. The treated tissues showed a mean viability >50%, indicating a non-irritating effect. The experiment was conducted on human-derived epidermal keratinocytes reconstructed in a three-dimensional structure, mimicking human epidermal tissue. The cell culture medium was assayed for the concentration of IL-18 cytokine and the cells were collected for MTT assays to evaluate their viability. Error bars represent the standard deviation of three independent experiments.

Table S1. Nitrogen adsorption isotherm data for pSiNP-OH, OxpSiNP and BAS-OxpSiNP samples.

	pSiNP-OH	OxpSiNP	BAS-OxpSiNP
**Surface area, S_{BET} (m^2/g)[a]**	359.9 ± 9.5	222.9 ± 4.2	143.3 ± 24.1
**Pore volume, V_{Pore} (cm^3/g)[b]**	0.99 ± 0.03	0.83 ± 0.08	0.76 ± 0.15
**Pore size, D_{Pore} (nm)[b]**	13.0 ±0.8	19.0 ± 1.5	23.2 ± 4.4

[a]Measured by nitrogen adsorption and determined using BET (Brunnauer–Emmett–Teller) analysis of the adsorption isotherms. [b]Measured by nitrogen adsorption and determined using BJH (Barrett–Joyner–Halenda) analysis of the adsorption/desorption isotherms.

Table S2. Hydrodynamic size and zeta potential of the indicated samples in HEPES buffer (50mM, pH7.4 with 0.1 mM $CoCl_2$) using dynamic light scattering (DLS). The "±" indicates the standard deviation of three independently prepared samples.

	Average Hydrodynamic Diameter (nm)	Zeta-Potential (mV)
pSiNP-OH	328.3 ± 42.3	-19.9 ± 1.3
PTE@pSiNP-OH	338.3 ± 42.6	-25.0 ± 0.6
OxpSiNP	220.7 ± 1.6	-31.5 ± 1.7
PTE@OxpSiNP	233.3 ± 8.2	-25.5 ± 3.4
BAS-OxpSiNP	306.7 ± 9.7	40.0 ± 3.1
PTE@BAS-OxpSiNP	756.3 ± 32.2	15.5 ± 1.8

Table S3. Enzyme mass loading (%) and estimated kinetic parameters of the immobilized PTE constructs and free PTE (L7ep3a variant).

Effect of Surface Chemistry on Kinetics						
Sample	Enzyme Mass Loading (%)*	Volume Loading ($mg{\cdot}cm^{-3}$)	V_{max} (μM/s)	k_{cat} (s^{-1})	K_m (μM)	k_{cat}/K_m ($M^{-1}s^{-1}$)
**PTE@OxpSiNP(L)**	5.7 ± 0.6	72.3	8.6 ± 0.5	315.4 ± 18.6	378.7 ± 29.3	$8.3\times10^5 \pm 4.8\times10^4$
**PTE@BAS-OxpSiNP**	4.0 ± 0.4	55.3	4.0 ± 0.7	144.8 ± 26.3	466.0 ± 94.2	$3.1\times10^5 \pm 2.3\times10^4$
**PTE@pSiNP-OH**	5.3 ± 1.2	56.6	0.9 ± 0.2	32.6 ± 7.0	85.8 ± 22.6	$3.8\times10^5 \pm 4.8\times10^4$
**Free PTE L7ep3a**	–	–	4.5 ± 1.0	163.4 ± 36.9	216.6 ± 65.2	$7.7\times10^5 \pm 1.2\times10^5$

Effect of Mass Loading of Enzyme in OxpSiNP on Kinetics						
Sample	Enzyme Mass Loading (%)*	Volume Loading ($mg{\cdot}cm^{-3}$)	V_{max} (μM/s)	k_{cat} (s^{-1})	K_m (μM)	k_{cat}/K_m ($M^{-1}s^{-1}$)
**PTE@OxpSiNP(L)**	5.7 ± 0.6	72.3	8.6 ± 0.5	315.4 ± 18.6	378.7 ± 29.3	$8.3\times10^5 \pm 4.8\times10^4$
**PTE@OxpSiNP(M)**	10.0 ± 0.5	132.5	6.5 ± 0.2	238.0 ± 9.1	270.1 ± 25.5	$8.8\times10^5 \pm 1.7\times10^4$
**PTE@OxpSiNP(H)**	15.4 ± 3.6	219.3	5.2 ± 0.4	190.6 ± 14.9	212.1 ± 33.3	$9.0\times10^5 \pm 6.8\times10^4$

*To determine the enzyme loading (wt%) in the nanoparticle constructs, the supernatant solutions were collected after the enzyme immobilization step by successive centrifugation and redispersion three times. Protein concentration in each collected supernatant was measured from a 2 μL aliquot by its absorbance at 280 nm using a Nanodrop 2000 (Thermo Fisher Scientific) absorbance spectrophotometer. For each supernatant sample, the measurements were repeated three times and the enzyme concentration present in each supernatant was calculated by average of the three measurements. The mass of enzyme in the three supernatants was added up and the enzyme loading was calculated based on the following equation:

$$\text{Eq: Mass Loading (\%)} = \frac{W_t - W_s}{(W_t - W_s) + W_p} \times 100$$

where W_t is the total mass of enzyme added to the loading process, W_s is the mass of unloaded enzyme present in the three supernatants and W_p is the mass of nanoparticles used in the immobilization process.

Table S4. Secreted IL-18 (pg/mL) from human-derived epidermal tissues and the stimulation index (SI)* following 24-hour exposure to the test materials.

		Tissue 1		Tissue 2	
**Sample**	**Concentration (mg/mL)**	**Secreted IL-18 (pg/mL)**	**SI**	**Secreted IL-18 (pg/mL)**	**SI**
PTE@OxpSiNP	10	3.0	1.3	3.7	1.0
	3	2.6	1.1	5.6	1.5
	1	5.2	2.3	3.7	1.0
	0.3	1.9	0.8	4.8	1.3
	0.1	1.9	0.8	1.6	0.4
	0.03	1.9	0.8	2.3	0.6
	0.01	1.9	0.8	2.3	0.6
	0.003	2.6	1.1	3.0	0.8
Untreated Tissues		3.7	1.6	2.6	0.7
H_2O Control		2.3	1.0	3.7	1.0
2% LA (Negative Control)		3.0	1.3	7.2	1.9
0.15% DNCB (Positive Control)		27.7	12.0	31.3	8.5

*The stimulation index (SI) is calculated as the ratio of secreted IL-18 in response to treatment with each test material divided by that of the H_2O control.
